# Genetic diversity and virulence variability of *Sclerotinia sclerotiorum* in Eastern and Northeastern India

**DOI:** 10.1371/journal.pone.0312472

**Published:** 2024-11-25

**Authors:** Tasvina R. Borah, Subrata Dutta, Ashis Roy Barman, Sujit Kumar Ray

**Affiliations:** 1 Department of Plant Pathology, Bidhan Chandra Krishi Viswavidyalaya, Mohanpur, West Bengal, India; 2 Department of Plant Pathology, RRS (CSZ), Bidhan Chandra Krishi Viswavidyalaya, Kakdwip, India; Universitat Jaume 1, SPAIN

## Abstract

*Sclerotinia sclerotiorum*, the necrotrophic cosmopolitan fungus, has become an emerging and re-emerging pathogen in the subtropical regions. Genetic diversity of 36 isolates of the fungus isolated from infected samples collected from the eastern and North eastern states was carried out using UP-PCR and SSR. Virulence variability was analysed based on four different measures. Among the eight UP-PCR primers and various combinations used, L-21, 3–2 and AA2M2-AS4 generated maximum number of fingerprints (13, 13 and 12, respectively) ranging from 100bp to 1kb. The isolates exhibited varied level of aggressiveness; majority (77.78%) were moderately virulent, 8.33% (22.22% of Assam and 6.67% of West Bengal) isolates were highly virulent, and 13.89% were less virulent. Several amplification products *viz*., 500bp generated by AA2M2-AS4, 150bp by AA2M2-L-21 and 100bp by L-21-3-2 were positively correlated with disease severity grading at 5% level of significance, whereas, 600bp band generated by AA2M2-3-2 was correlated at 1% level of significance. This indicates presence of these bands in highly virulent isolates. Out of the eight SSR primers, TATG9 did not generate any amplification and the isolates were divided into two major groups; the group II contained single isolate from Nagaland (NG4) indicating it to be genetically diverse from rest of the isolates. The subgroup A of the major group I was the largest and most diverse group with 11 members indicating genetic admixture within different geographic populations with different levels of similarity (70–100%). Genetic diversity based on SSR banding pattern showed highest value of Nei’s gene diversity and Shannon’s index of diversity (%pb = 61.11; h = 0.219; I = 0.330) for the Nagaland population with 9 members followed by West Bengal population with 15 members. Nei’s genetic distance of all the tested populations was low, ranging from 0.0014 to 0.2350; however, genetic identity was high ranging from 0.7905 to 0.9986. The findings suggest that the pathogen populations of eastern and North eastern region were predominantly clonal with some evidence of infrequent out crossing.

## Introduction

Disease incited by *Sclerotinia sclerotiorum* (Lib.) de Bary, is known by many names [1, 2] including cottony rot, watery soft rot, stem rot, drop, crown rot, blossom blight and perhaps most common, white mold. A necrotrophic and homothallic ascomycete, the fungus reproduces asexually through sclerotia and sexually through ascospores produced by self-fertilization. An important plant pathogen with broad ecological distribution, it infects wide range of hosts including many important field and vegetable crops. The fungus infects more than 500 species of host plants [3] and has become the reason of increasing concern with its increasing host range over time [4, 5].

Earlier, the fungus was mostly recorded from cool and moist parts of the globe [1, 6], but new reports suggest significant level of occurrence in the hot as well as dry areas, because of its genetic and adaptive shift [7]. The pathogen, thus, has wide geographical distribution and several of its biological features make it a highly threatening pathogen to diverse economic crop plants across the world.

Prominent morphological variations of the fungus coupled with molecular variation based on various molecular tools indicated wide range of variability [6–8] and established high level of intraspecific phenotypic and genetic variability. *S*. s*clerotiorum* population is mainly clonal under subtropical climatic conditions while sexual reproduction could be seen in the population of temperate climate areas [9, 10]. *S*. *sclerotiorum* isolates are also known to have exhibited significant variations in aggressiveness or the severity of disease on various hosts [8, 11, 12].

Crop production in the country is known to be affected by *S*. *sclerotiorum* for several decades but the impact is felt much more in recent years especially in the subtropical zones, probably with the emergence of diverse virulent forms along with changes in the climatic conditions towards more favourable proportions [4]. Despite the substantial impact on agricultural production in the entire eastern and North eastern regions, little is known about the variations within this important pathogen isolated from various hosts. Management of the pathogen becomes arduous due to its long survivability, wide host range and lack of resistance sources. Studies on the variability of *S*. *sclerotiorum* will help in understanding the genetic diversity of the pathogen population in the region, epidemiological aspects of the disease, management with fungicides and resistant cultivars. Genetic variations within and among the pathogen populations is imperative to formulate strategic implementation of the combination of management techniques [13]. The present study was conducted to assess the molecular and pathogenic variability of *S*. *sclerotioum* on different crops in the North eastern and eastern region of the country.

## Materials and methods

### Isolation, purification and identification of *S*. *sclerotiorum* isolates

Surveys were conducted during winter months (November–February) for three consecutive years starting from 2015–16 to collect *S*. *sclerotiorum* infected parts of various hosts from four North eastern states *viz*., Assam, Mizoram, Nagaland, Sikkim and the eastern state of West Bengal encompassing hot subhumid to humid Eco-region (Assam and Bengal plain), warm perhumid Eco-region (Eastern Himalayas) and warm perhumid Ecoregion (North-Eastern Hills [Purvanchal]).

Diseased plant parts, *i*.*e*., fruits, stem, leaves and inflorescence of various hosts showing typical symptoms along with sclerotia were collected maintaining aseptic condition. The diseased plant tissue (cut into 1 to 1.5 cm length bits/pieces) or individual sclerotium were surface sterilized with 1 percent sodium hypochlorite solution for 1 minute, washed three times with sterile distilled water and blot dried. These bits or single sclerotium were then placed on Petri dish containing PDA medium and incubated at 22±2°C for 2 to 3 days. Pure culture of the fungus was obtained by hyphal tip culture after 2–3 days of incubation. The pure cultures were maintained in refrigerator at 4°C with periodical sub-culturing at monthly intervals. Morphological and biochemical characterization of the isolates were done by Borah et. al. [5] to study the phenotypic variability and pathogenic diversity amongst the isolates.

### Virulence test

*In vitro* assessment of virulence of the fungal isolates was performed on the basis of average lesion length (AvLL) with the mycelial plug inoculation technique in harvested French bean fruits in incubator and French bean plants (cv. Falguni) under growth chamber conditions. To determine variations of virulence of the isolates, three French bean fruits (considering each as a replicate) were inoculated for each isolate with 2 mm of mycelial plug and incubated at 22±2°C. Likewise, 30 days old plants were artificially inoculated (in three replications) with 2 mm of mycelial plug. Temperature of 25°C and relative humidity (RH) of 85% were maintained in the growth chamber. Lesion lengths were measured at 24h interval from 3 days after inoculation and up to 5 days for each of the French bean fruit, and data obtained was averaged to get AvLL for each isolate. Similarly, the lesion lengths were measured from 7 days after inoculation for each plant up to 10 days. Disease grading was performed as per Modified Petzoldt and Dickson scale of 1–9 [14]. Further, area under lesion progress curve (AULPC) from the lesion length on French bean fruit and area under disease progress curve (AUDPC) from the measurements of the necrotic lesions on French bean plants were calculated to know the aggressiveness of the isolates.

### DNA extraction

Genomic DNA of *S*. *scleortiorum* isolates was extracted with Promega Wizard SV Genomic DNA Purification System (A2360) according to the manufacturer’s protocol. The purified genomic DNA was quantified by loading 2μl of DNA onto spectrophotometer (Thermo Scientific NanoDropTM 1000). The measurement of A260/A280 indicated the quality of the purified DNA. The genomic DNA was also run in 0.8% agarose gel electrophoresis stained with ethidium bromide.

### PCR amplification of ITS regions

PCR amplification of Internal Transcribed Spacers (ITS) regions was performed using universal primers ITS-1 and ITS-4 [15] in Thermalcycler (Thermo Fisher Scientific). Amplification was carried out in a volume of 25 μl reaction mixture with 1.5 μl of 10× Taq buffer containing 15 mM MgCl_2_, 1.0 μl of ITS1 primer (5 pM/μl), 1.0 μl of ITS-4 primer (5 pM/μl), 1 μl of 2.5 mM dNTP mix each, 0.5 U of Taq polymerase (5 U/μl) (Promega) and 3 μl (20–30 ng/μl) of DNA sample. PCR carried out with 35 cycles, were denaturation at 94°C for 50 sec, annealing at 56°C and extension at 72°C for 1 min each with initial denaturation at 94°C for 5 min and final extension at 72°C for 3 min. Amplified PCR products were visualised in 1 per cent agarose gel in 1× TAE buffer, compared with 1 kb DNA ladder (GeNei)under gel document unit (BioRad, USA) with ethidium bromide staining.

#### Sequencing of ITS regions and sequence analysis

Direct sequencing of the PCR products was conducted for the positive strand in forward direction using Applied Biosystems sequencers 373A and 377. Sequencing reaction was conducted by Sci Genom Labs Pvt. Ltd, Cochin, Kerala, India. The 36 sequences of the ITS regions of *S*. *sclerotiorum* isolates were deposited at NCBI nucleotide database through Gen Bank Bankit submission tools and accession numbers were obtained.

### Phylogenetic analysis

ITS 1, partial sequence; 5.8S ribosomal RNA gene and ITS 4, complete sequence; and 28S ribosomal RNA gene, partial sequence of 42 isolates comprising the three *Sclerotinia* species namely *S*. *sclerotiorum*, *S*. *minor* and *S*. *trifoliorum* infecting different hosts and from different geographical regions of world were retrieved from NCBI: GenBank (http://www.ncbi.nlm.nih.gov/) (S1 Table). ClustalW multiple sequence alignment of target sequences and retrieved sequences, trimming and generation of phylogenetic tree using Neighbour-Joining algorithm was performed using MEGA7 program [16].

### Variability with molecular markers

#### UP-PCR

Eight universal primers (Table 1) were used in single or in pairwise combinations for studying molecular variability of the 36 *S*. *sclerotiorum* isolates. UP-PCR amplification was performed in 25μl reaction mixture containing 5 μl 5× PCR buffer (Promega), 2.0 μl MgCl_2_ (25 mM), 1.0μl dNTPs (2.5 mM each), 1.0 μl of each primer (100 ng/μl), 0.35 μl Taq DNA polymerase (Promega) and 3.0 μl template DNA (50 ng/μl). PCR tubes were placed in Thermalcycler (Thermo Fisher Scientific) for an initial denaturation at 94°C for 3 min followed by 35 cycles of 50 sec at 94°C (denaturation), 1min 10 sec at the optimal temperature for each primer (annealing), 1 min at 72°C (extension) and final extension at 72°C for 3 min.

**Table 1:** Universal primers used for performing UP-PCR of the *S*. *sclerotiorum* isolates.

Primer name	Sequence (5’-3’)	Annealing temperature(°C)
ITS 4 [15]	TCCTCCGCTTATTGATATGC	56
ITS 6 [15]	GAAGGTGAAGTCGTAACAAGG	56
AA2M2 [17]	CTGCGACCCAGAGCGG	56
AS 4 [17]	TGTGGGCGCTCGACAC	56
3–2 [18]	TAAGGGCGGTGCCAGT	56
AS15in [18]	CATTGCTGGCGAATCGG	57
AS 15[18]	GGCTAAGCGGTCGTTAC	55
L—21 [19]	GGATCCGAGGGTGGCGGT T	55

PCR amplified products were subjected to 1% horizontal agarose gel electrophoresis along with 1 kb and 100 bp DNA ladder. Ethidium bromide (1.5 μl of stock solution in 100 ml agarose gel) was used to stain the gel and the gels were documented using Gel document unit (BioRad, USA).

#### Microsatellites

Eight primer sets were selected (Table 2) from the set of SSR primers developed by Sirjusingh and Kohn [20]. PCR amplification was performed in 25μl reaction mixture containing 11.7μl sterile double distilled water, 5μl 5× PCR buffer (Promega), 2.0μl MgCl_2_ (25 mM), 1.0μl dNTPs (2.5 mM each), 1.0μl of forward and reverse primer each (100 ng/μl), 0.35μl Taq DNA polymerase (Promega) and 3.0μl template DNA (50 ng/μl). PCR reaction was carried out in a Thermalcycler (Thermo Fisher Scientific) with the following reaction cycles: initial denaturation at 94°C for 3 min followed by 35 cycles of 50 sec at 94°C (denaturation), 1min 10 sec at the optimal temperature for each primer set (annealing), 1 min at 72°C (extension); final extension at 72°C for 3 min and was followed by hold at 4°C.

**Table 2:** List of microsatellite primers used for variability study.

Locus	Repeat Motif	Primer sequence	Size range (bp)	Annealing temperature (°C)
7–2	(GA)_14_	TTTGCGTATTATGGTGGGC	160–172	56
		ATGGCGCAACTCTCAATAGG		
12–2	(CA)_9_	CGATAATTTCCCCTCACTTGC	215–225	59
		GGAAGTCCTGATATCGTTGAGG		
17–3	(TTA)_9_	TCATAGTGAGTGCATGATGCC	345–390	57
		CAGGGATGACTTTGGAATGG		
55–4	TACA_10_	GTTTTCGGTTGTGTGCTGG	173–221	58
		GCTCGTTCAAGCTCAGCAAG		
92–4	(CT)_12_	TCGCCTCAGAAGAATGTGC	374–378	58
		AGCGGGTTACAAGGAGATGG		
106–4	(CATA)_25_	TGCATCTCGATGCTTGAATC	49–571	57
		CCTGCAGGGAGAAACATCAC		
110–4	(TATG)_9_	ATCCCTAACATCCCTAACGC	362–378	56
		GGAGAATTGAAGAATTGAATGC		
114–4	(AGAT)_14_(AAGC)_4_	GCTCCTGTATACCATGTCTTG	351–391	56
		GGACTTTCGGACATGATGAT		

PCR products were separated by horizontal gel electrophoresis using 2% agarose gel in 1× TAE buffer at 75 V for 45 min using Biotech standard gel electrophoresis units, where 100 bp DNA ladder were used for comparison.

### Statistical analysis

Rank analysis and cumulative ranking of the isolates based on lesion length, AULPC, grading and AUDPC parameters were performed in Microsoft Excel for comparing the virulence of the isolates.

#### Scoring and analysis of marker data

The data set of DNA markers obtained by scoring the presence of bands as 1 and absence as 0 from all the isolates was analysed with UPGMA by the NTSYSpc 2.0 software and dendogram was constructed using Jaccard’s coefficient. Genetic diversity analysis of the geographical populations (isolates from a single state was considered as one geographical population) was studied with POPGENE 1.32 software. Genetic differentiation within and among populations was estimated by analysis of molecular variance (AMOVA) using the poppr version 2.2.0 of R [21] with 1000 permutations with clone correction.

For the identification of genetic relationships among individuals and populations through population structure analysis, the population data were further analysed using the program STRUCTURE 2.3.4 with recommended admixture model and correlated allele frequencies to check for hidden genetic structure in the populations.

## Results

### Survey, isolation and characterization of *S*. *sclerotiorum* isolates

Thirty-six isolates of the fungus were isolated from 28 infected plant species from the five different widely spaced geographical locations, *viz*., Assam, Mizoram, Nagaland, Sikkim and West Bengal. Pure cultures of each of the isolates (in duplicate) were deposited to the National Agriculturally Important Microbial Culture Collection (NAIMCC) of ICAR National Bureau of Agriculturally Important Microorganisms, located at Mau Nath Bhanjan, Uttar Pradesh and accession numbers for all the 36 isolates were obtained (S2 Table). This authenticated the fungal isolates to be *S*. *sclerotiorum*.

### Virulence test

Significant variation in virulence of the thirty-six *S*. *sclerotiorum* isolates was observed. Isolates were categorized into three virulence groups, *viz*., low virulent (LV), moderately virulent (MV) and highly virulent (HV) on the basis of the AvLL, grading, AULPC and AUDPC (S3 Table). The AvLL data indicates 11.11% (AS4, AS6, WB1 and WB7) and AULPC data indicates 5.56% (AS4 and WB1) of the isolates were categorized under highly virulent group. As per AvLL and AULPC, 66.67% and 41.67% of the isolates belonged to moderately virulent group, respectively. Disease grading of the Frenchbean plants assigned 75.00% of the isolates as highly virulent and rest of the isolates as moderately virulent, however, none of the isolates could be assigned in low virulent category. According to AUDPC, 13.89%, 77.78% and 8.33% of the isolates were less, moderate and highly aggressive, respectively. Highly aggressive isolates were AS4, AS6 and WB1.

**Fig 1:**
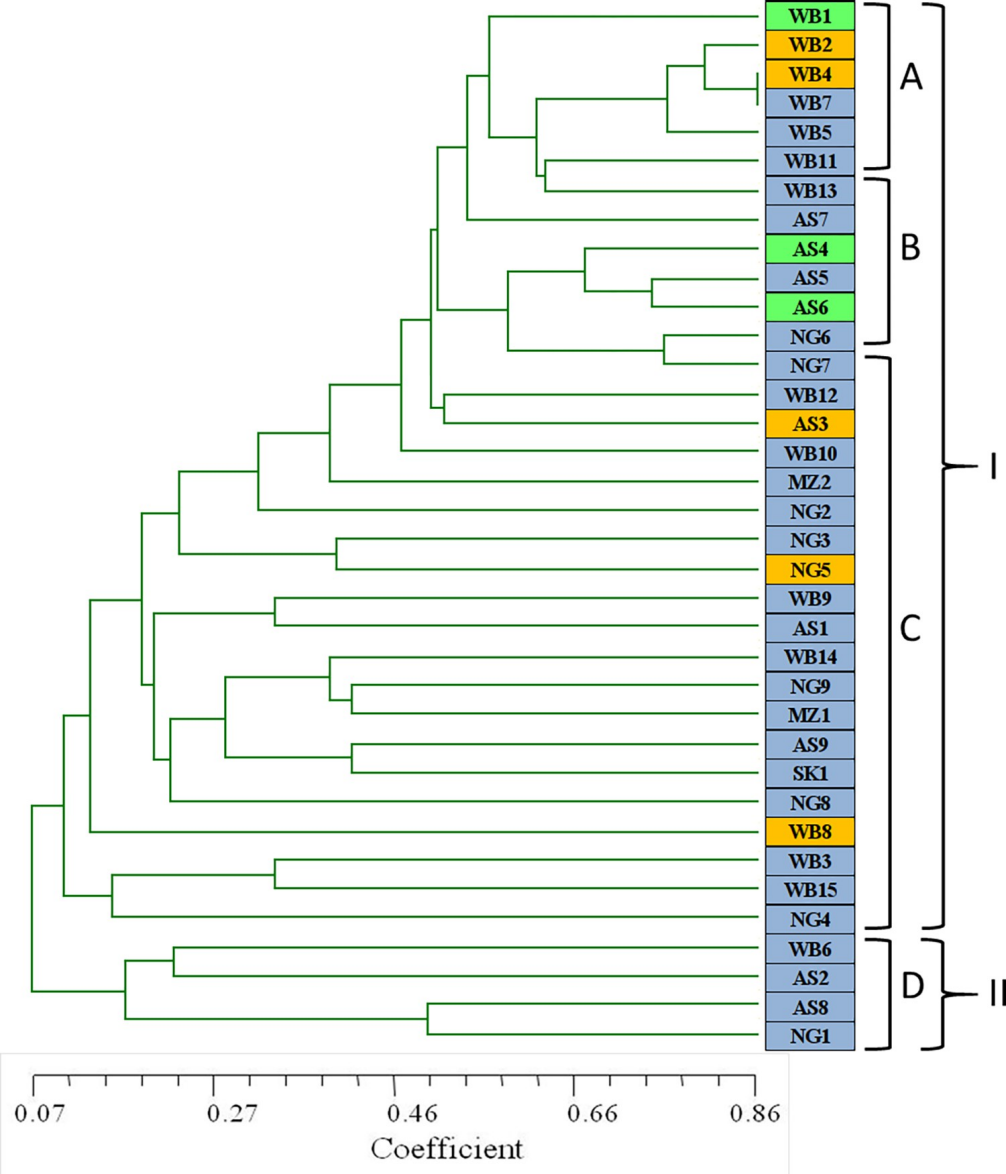
UP-PCR based dendrogram of *S*. *sclerotiorum* isolates, coloured according to the pathogenicity of the isolates as- green colour indicate highly virulent isolates whereas blue and yellow colours show moderate and low virulent isolates, respectively.

Rank analysis and cumulative ranking of the isolates combining AvLL, AULPC, grading and AUDPC parameters were performed to classify isolates on the basis of virulence (S4 Table). Cumulative ranking of the isolates indicated highest rank of 35, which was assigned to the three highly virulent isolates AS4, AS6 and WB1, whereas the lowest rank like 1, 2 and 4 were assigned to the low virulent isolates NG5, WB8 and WB2, respectively. The results revealed that majority (88.89%) of the West Bengal isolates (WB3, WB5, WB6, WB7, WB9, WB10, WB11, WB12, WB13, WB14 and WB15) were moderately virulent, only one (2.78%) isolate (WB1) was highly virulent and the rest (8.33%) of the isolates (WB2, WB4 and WB8) were less virulent. The 22.22% of the Assam isolates (AS4 and AS6) were highly virulent, 66.67% (AS1, AS2, AS5, AS7, AS8 and AS9) were moderately virulent and 11.11% (AS3) were less virulent. Similarly, all the Nagaland isolates (88.89%) were moderately virulent except one (NG5), which was low virulent. The results thus indicated significant pathogenic diversity among the isolates irrespective of their geographical location and host of origin (Fig 1).

### Molecular identification of *S*. *sclerotiorum* isolates

Molecular identification was performed based on ITS sequence homology. Genomic DNA of thirty-six isolates of *S*. *sclerotiorum* was isolated and PCR amplification was performed with internal transcribed spacer (ITS) specific primers (ITS1 and ITS4) which yielded single DNA fragment of approximately 550 bp for all the 36 isolates (S1 Fig). The amplified ITS regions were sequenced and nucleotide BLASTN revealed maximum identity score of 99–100% with *S*. *sclerotiorum* accessions from other parts of India and world (S5 Table). Nucleotide sequences of the isolates were deposited to NCBI GenBank database to obtain accession numbers (S2 Table).

#### Evolutionary relationship

The 36 ITS sequences of *S*. *sclerotiorum* considered in the current study were aligned among themselves. The isolates depicted 99.7–100% similarity among themselves with single nucleotide variation in WB5 and WB12 isolates from West Bengal. Twelve representative isolates belonging to the eastern and different north-eastern states were selected from the 36 isolates for further alignment with other 52 number of nucleotide sequences belonging to *S*. *sclerotiorum*, *S*. *trifoliorum* and *S*. *minor* from India and World. All the twelve isolates under this study were grouped in Cluster A with all the other *S*. *sclerotiorum* from India and World and *S*. *minor* sequences, indicating common ancestral origin (S2 Fig). It was evident that the twelve isolates were also highly similar to other Indian isolates of *S*. *sclerotiorum* indicating low genetic variability of the clonal population of the fungus in the eastern and north-eastern regions of India. The similarity with world isolates of *S*. *sclerotiorum* ranged from 97.3–100.0% (S6 Table). However, *S*. *minor* formed a separate sub-cluster within Cluster A. Four of the *S*. *trifoliorum* sequences formed a separate sub-cluster within Cluster A. In Cluster B represented a single isolate of *S*. *trifoliorum* away from other isolates of the same species and other *Sclerotinia* sp. Two Canadian isolates of *S*. *sclerotiorum* separated out from all the isolates of *Sclerotinia* and formed a separate group, Cluster C (S2 Fig).

#### Phylotyping by UP-PCR

Among the eight UP-PCR primers, four primers, *viz*., AA2M2, AS4, 3–2 and L-21 have generated fingerprints with a relatively high number of bands individually and in combination, for most of the isolates. Maximum numbers of bands (13) for most of the isolates were generated by the primers L-21 and 3–2 followed by primer combination AA2M2-AS4 with 12 number of bands. The minimum and maximum size of amplicons generated by these primers and the primer combinations was 100bp and 1kb, respectively (S3 Fig). A dendrogram was generated by SAHN subroutine clustering using Jaccard coefficient as presented in Fig 1. Similarity coefficient matrix of *S*. *sclerotiorum* based on UPGMA method showed similarity coefficient of 86.15% among all the isolates (S7 Table). The isolates were classified into two major clusters with several sub-clusters. The cluster IA contained seven West Bengal isolates (WB1, WB2, WB4, WB7, WB5, WB11 and WB13) with 60–80% similarity. In the IB cluster four Assam (AS7, AS4, AS5 and AS6) and two Nagaland (NG6 and NG7) isolates were grouped together with 60–70% similarity. The IIA cluster with four isolates (WB6, AS2, AS8 and NG1) was diverged from all other isolates at 7% similarity.

UP-PCR based clustering of the isolates was not found to be associated with virulence level of the isolates (Fig 1). This indicates the fact that isolates belonging to the same group could show great diversity, which might result in apparent difference in their virulence level. Pearson correlation matrix distinguished specific bands significantly correlated with the pathogenicity (grading and AUDPC) of *S*. *sclerotiorum* isolates on French bean plants (Table 3). The amplicon products 500bp, 150bp and 100bp generated by the primers AA2M2-AS4, AA2M2-L-21 and L-21-3-2, respectively, were positively correlated with disease severity grading at 5% level of significance whereas 600bp product generated by AA2M2-3-2 was correlated at 1% level of significance, indicating presence of these bands in highly virulent isolates of *S*. *sclerotiorum*.

**Table 3:** Correlation studies of pathogenicity of different *S*. *sclerotiorum* isolates with UP-PCR gel bands.

Primer combination	*UP-PCR product (bp) *	*Grading*		AUDPC	
		*Coefficient of correlation*	*p Value*	*Coefficient of correlation*	*p Value*
Primer 3–2	150bp	0.284	0.143	0.201	0.304
Primer AA2M2-AS4	500bp	0.494*	0.032	0.399	0.091
Primer AA2M2-3-2	600bp	0.576**	0.006	0.391	0.080
	700bp	0.331	0.143	0.307	0.176
Primer AA2M2-L-21	150bp	0.406*	0.032	0.260	0.181
	600bp	0.375	0.049	0.249	0.201
Primer L-21-3-2	100bp	0.373*	0.043	0.245	0.193

Genetic diversity analysis of the geographical population using POPGENE package version 1.32 revealed overall estimate of polymorphic bands for the five populations as 89. Population diversity was highest for the isolates collected from West Bengal in terms of percentage of polymorphic bands, Nei’s gene diversity and Shannon’s index of diversity (%pb = 87.64; h = 0.369; I = 0.531). Diversity for Sikkim population was not observed because only one member was obtained. Diversity was also low for the Mizoram population (%pb = 34.83; h = 0.174; I = 0.241) constituted with only two members (Table 4). The Assam and Nagaland population with same number of isolates varied in the degree of population diversity. Shannon’s diversity index (I) was comparatively higher for the Assam population (0.482) while it was 0.395 for Nagaland population.

**Table 4:** Molecular variance analysis of five *S*. *sclerotiorum* geographic populations with UP-PCR.

Population	Population size	na	ne	h	I	pb	% pb
West Bengal	15	1.876	1.677	0.369	0.531	78	87.64
Assam	9	1.820	1.595	0.331	0.482	73	82.02
Nagaland	9	1.708	1.463	0.267	0.395	63	70.79
Mizoram	2	1.348	1.348	0.174	0.241	31	34.83
Sikkim	1	1.000	1.000	0.000	0.000	0	0.00
SD		0.000	0.310	0.136	0.165		

Note: na-average number of alleles; ne-effective number of alleles; h-Nei’s gene diversity; I-Shannon’s diversity index; pb-polymorphic bands; %pb-percent polymorphic bands; SD-standard deviation

Data presented in Table 5 signifies the maximum genetic identity (0.960) and minimum genetic distance (0.0408) between West Bengal and Assam populations. According to Nei [22] this measure in genetic differences between the populations may arise due to mutation and genetic drift. Pair wise genetic identity of the other three populations was in the range of 0.839 to 0.949 and genetic distance was 0.0524 to 0.1745.

**Table 5:** Genetic identity (above diagonal) and genetic distance (below diagonal) among the geographical populations.

Pop. ID	WB	AS	NG	MZ	SK
**WB**	-	0.9600	0.8787	0.8876	0.7792
**AS**	0.0408	-	0.9490	0.9058	0.8564
**NG**	0.1293	0.0524	-	0.8924	0.9094
**MZ**	0.1192	0.0989	0.1139	-	0.8399
**SK**	0.2495	0.1550	0.0949	0.1745	-

**Fig 2:**
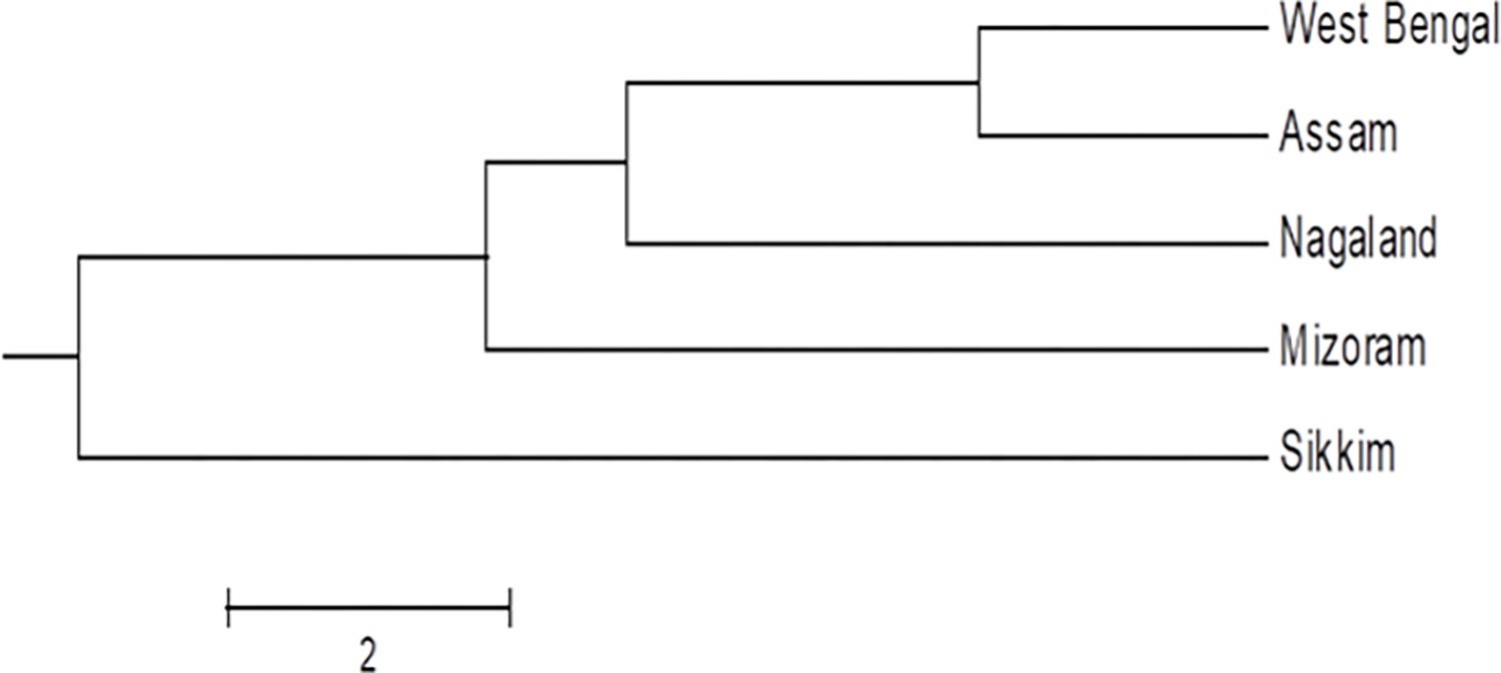
Polygenetic map of different geographic populations of *S*. *sclerotiorum* based on Nei’s coefficients and group average hierarchical clusters produced by the POPGENE 1.32.

Dendrogram obtained with Nei’s coefficient also depicted two major groups with a common origin; Sikkim isolate making up one of the major groups while the other four populations from Assam, Mizoram, Nagaland and West Bengal comprised subgroups of the other major group (Fig 2). Assam and West Bengal populations belonged to the same subgroup with similar genetic distance from the origin. Among the five geographical populations UP-PCR revealed that the variation within the populations is higher than that amongst the populations as is evident from Table 6.

**Table 6:** Molecular variance analysis within and among *S*. *sclerotiorum* populations for UP-PCR data (AMOVA).

Source of variation	Degrees of freedom	Sum of squares	Variance components	Percentage of variation
Among populations	4	89.289	0.981	5.72
Within populations	31	501.100	16.164	94.28
Total	35	590.389	17.145	100.00

Fixation Index FST: 0.05721

#### Phylotyping by SSR

**Fig 3:**
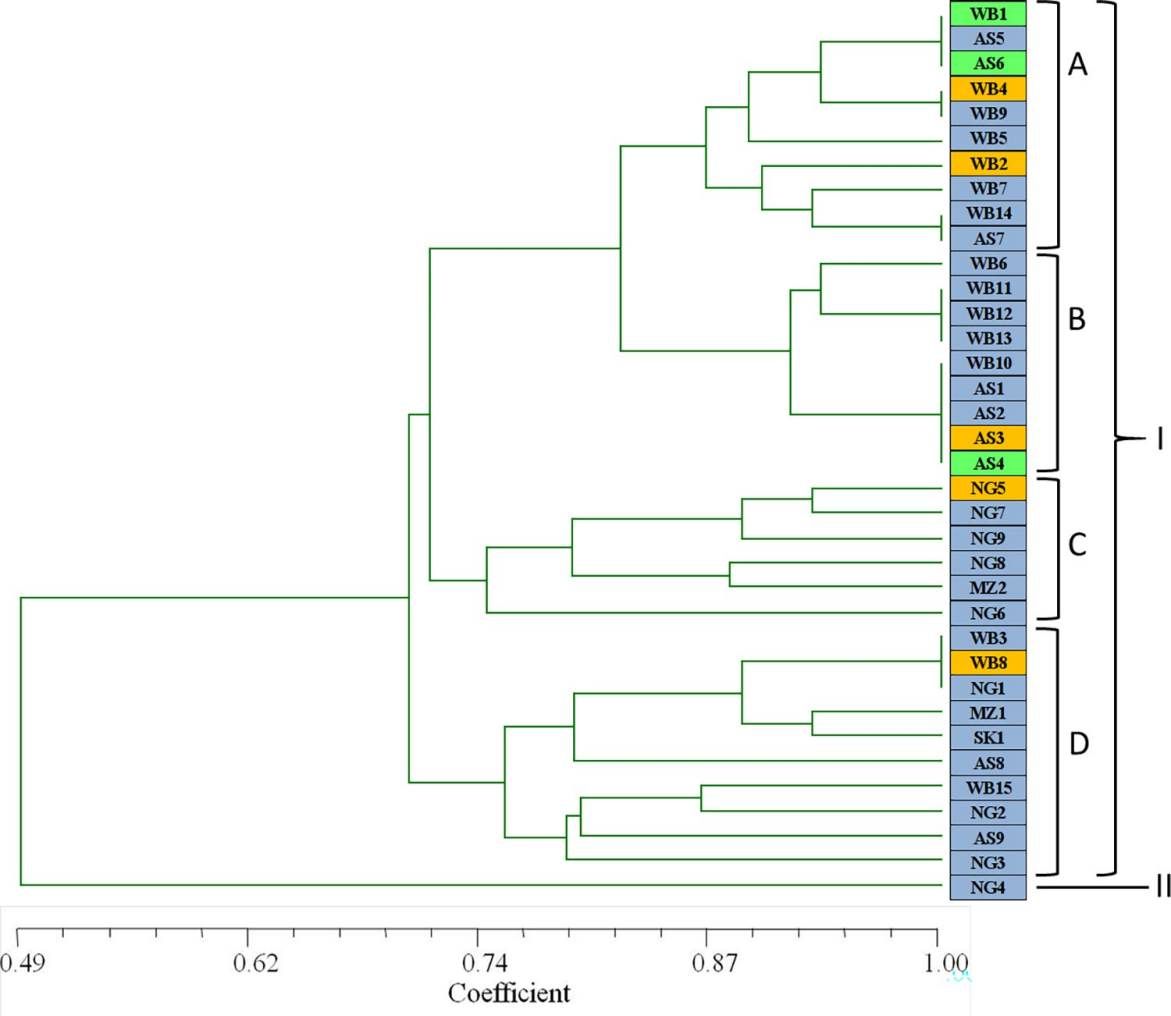
UPGMA dendrogram showing the genetic relationship between the 36 isolates of *S*. *sclerotiorum* based on SSR marker.

Out of the eight SSR primers (S4 Fig), TATG9 did not generate any amplification of the isolates. All the isolates were divided into two major groups based on SSR analysis (Fig 3); the group II contained single isolate from Nagaland (NG4) indicating it to be genetically diverse from rest of the isolates. The major group I was divided into four sub groups with different levels of similarity (70–100%). The first subgroup (A) was the largest and most diverse group with 11 members indicating genetic admixture within different geographic populations. The B, C and D subgroups included 6, 8 and 10 members, respectively. In the results of the present investigations, haplotypes obtained provided evidence of the presence of genetic diversity within the populations. However, results of cluster analysis suggested absence of relationship of genetic diversity among the populations with geographic origin or host type.

The percentage of polymorphic bands, the average number of alleles (na) and the number of effective alleles (ne) of the five geographical populations with different population size were found to be different. Genetic diversity of the geographical populations based on SSR banding pattern showed highest value of Nei’s gene diversity and Shannon’s index of diversity (%pb = 61.11; h = 0.219; I = 0.330) for the Nagaland population with 9 members followed by West Bengal population with 15 members (Table 7). The Assam population, although with same number of isolates as Nagaland population, was comparatively less diverse (%pb = 38.89; h = 0.115; I = 0.181). Among the Mizoram isolates, only two members exhibited diversity with 33.33% of polymorphic bands, Nei’s gene diversity of 0.167 and Shannon’s diversity index of 0.231. Total number of polymorphic bands were 14 and percentage of polymorphic bands was 77.78 for the populations. Gene differentiation (Gst) among the different populations was 0.3832 while gene flow was 0.8048, indicating that the gene permutation and interaction do exist within the tested isolates.

**Table 7:** Molecular variance analysis of five *S*. *sclerotiorum* geographic populations with SSR.

Population	Population size	na	ne	h	I	pb	% pb	Gst	Nm
West Bengal	15	1.500	1.266	0.161	0.246	9	50.00	0.3832	0.8048
Assam	9	1.389	1.183	0.115	0.181	7	38.89		
Nagaland	9	1.611	1.362	0.219	0.330	11	61.11		
Mizoram	2	1.33	1.33	0.167	0.231	6	33.33		
Sikkim	1	1.00	1.00	0.00	0.00	0	0.00		
SD		0.428	0.381	0.195	0.268				

Note: na-average number of alleles; ne-effective number of alleles; h-Nei’s gene diversity; I-Shannon’s diversity index; pb-polymorphic bands; %pb- percent polymorphic bands; Gst-coefficient of gene differentiation; Nm-coefficient of gene flow; SD-standard deviation

Nei’s genetic distance of all the tested populations was low (Table 8), ranging from 0.0014 to 0.2350, however, genetic identity was high ranging from 0.7905 to 0.9986. Nagaland and Mizoram populations showed closest genetic distance (0.0014) whereas Assam and Sikkim populations exhibited farthest distance (0.2350). Populations with the minimum genetic distance, *i*.*e*., distance among the Nagaland and Mizoram populations shared maximum genetic identity (0.9986) while Assam and Sikkim populations with maximum genetic distance showed minimum genetic identity (0.7905).

**Table 8:** Genetic identity (above diagonal) and genetic distance (below diagonal) among the geographical populations.

Pop. ID	WB	AS	NG	MZ	SK
**WB**	-	0.9871	0.9040	0.8970	0.8438
**AS**	0.0129	-	0.8948	0.8740	0.7905
**NG**	0.1010	0.1112	-	0.9986	0.8948
**MZ**	0.1087	0.1347	0.0014	-	0.9449
**SK**	0.1698	0.2350	0.1112	0.0567	-

**Fig 4:**
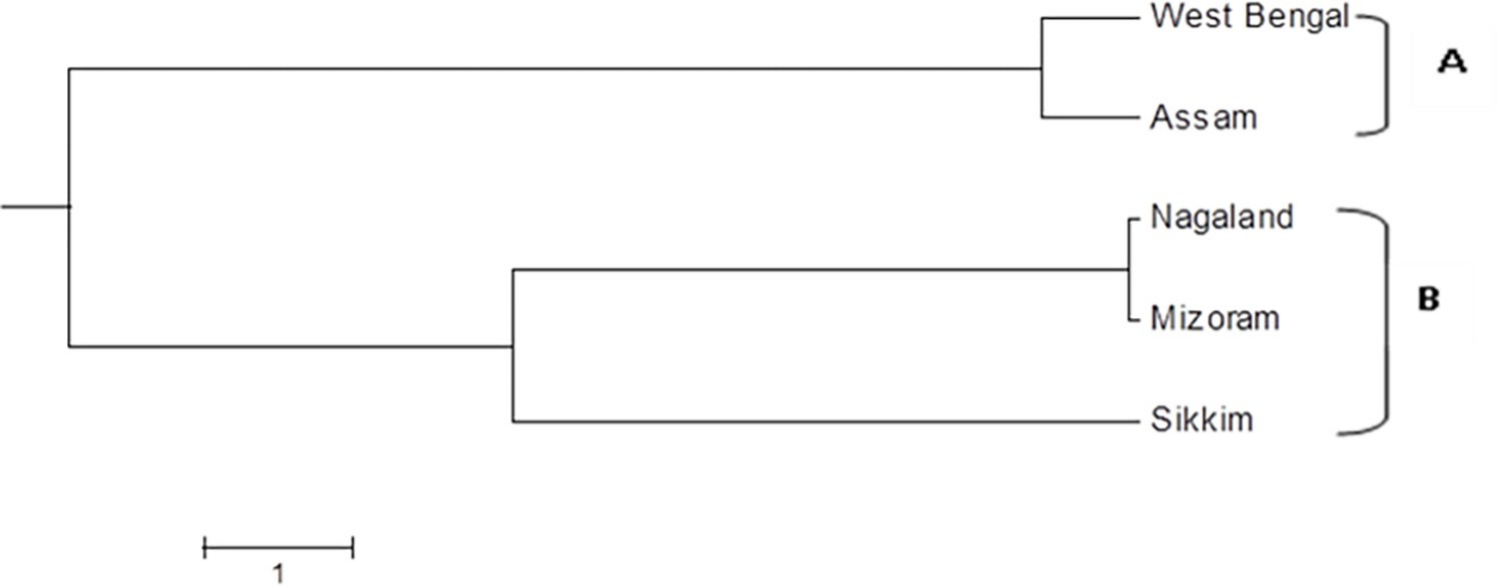
UPGMA polygenetic map of different *S*. *sclerotiorum* populations based on average hierarchical clusters produced by the POPGENE 1.32.

The matrix of Nei’s genetic distances suggested that the five tested populations could be divided into two distinct clusters (Fig 4). The cluster A included populations from West Bengal and Assam, indicating genetic closeness. Similarly, Nagaland and Mizoram populations had minimum genetic distance conversely sharing maximum genetic identity. Sikkim population showed closer genetic distance to Nagaland and Mizoram population compared with populations from West Bengal and Assam. Although geographic distance from Sikkim to Nagaland and Mizoram is much farther than distance between Sikkim and West Bengal or Assam, indicating geographic distance is not the key determinant for genetic diversity of the tested isolates.

Genetic differentiation within and among population estimates (Table 9) showed that genetic variations within populations was more (1.672) than among populations (0.405) with 31 and 4 degrees of freedom, respectively. Consequently, percent genetic variation within the five tested geographic populations was 80.52% while among populations percent genetic variation was 19.48% only.

**Table 9:** Molecular variance analysis within and among *S*. *sclerotiorum* populations for SSR data (AMOVA).

Source of variation	Degrees of freedom	Sum of squares	Variance components	Percentage of variation
Among populations	4	16.850	0.405	19.48
Within populations	31	51.844	1.672	80.52
Total	35	68.694	2.077	100.00

Fixation Index FST: 0.195

Significance tests

#### Population structure

**Fig 5:**
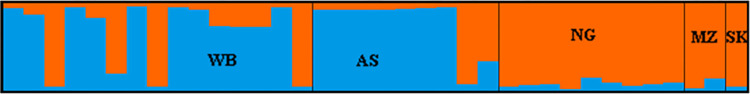
Distruct output by CLUMPAK representing admixture analysis of population structure for the isolates of *S*. *sclerotiorum*from West Bengal (WB), Assam (AS), Nagaland (NG), Mizoram (MZ) and Sikkim (SK).

Identification of genetically homogeneous groups of individuals in populations was detected by the true number of clusters (K) in STRUCTURE package. The results both prior to and following clone correction, supported two subpopulations (K = 2), with most individual isolates having probability of belonging to either subpopulation or having an admixture of the two genotypes (Fig 5). This suggests that the pathogen populations were predominantly clonal with some evidence of infrequent out crossing.

## Discussion

### Status of *S*. *sclerotinia* isolates from eastern and North eastern region

Thirty-six pathogenic fungal isolates obtained from different hosts and different agro-ecological zones of the four northeastern states and West Bengal, were identified as *S*. *sclerotiorum* by molecular detection using the amplification of around 550 bp ITS regions and sequencing of the same. Manjunatha et al. [7] found no variability among nucleotide sequences of ITS regions as well as with ITS-RFLP for 9 isolates of *S*. *sclerotiorum* collected from different hosts and geographical locations of India. The isolates were found to be virulent with varied level of aggressiveness. Among all the isolates, 8.33% of the isolates (22.22% of Assam and 6.67% of West Bengal) were highly virulent, 77.78% were moderately virulent and 13.89% were less virulent. Thus, maximum numbers of the isolates were moderately virulent followed by the number of the less virulent and highly virulent isolates. The present investigation was in accordance with that of Zancan et. al. [23] who obtained three groups for 25 isolates based on their aggressiveness as was evident from straw test rating (Modified Petzoldt and Dickson scale) and t grouping. *S*. *sclerotiorum* isolates were also categorized into most aggressive, intermediately aggressive and least aggressive based on significant differences in pathogenicity of the isolates regardless of the isolate origin [24]. Lesion length scoring system was adopted [25] to categorize 64 isolates of *S*. *sclerotiorum* into five groups based on virulence pattern of the isolates. Variation in virulence of isolates of *S*. *sclerotiorum* was also reported by several other workers [26, 27].

### Genetic diversity and virulence variability of the isolates

The present results clearly support existence of low genetic variability among the *S*. *sclerotiorum* populations in the eastern and northeastern part of the country. The present findings were in agreement with the observation of several earlier research [7, 28, 29]. The evolutionary history of the twelve isolates considered in this study (representative of each of the five states) along with 40 other ITS sequences depicted the same ancestral origin with other *S*. *sclerotiorum*, *S*. *trifoliorum* and *S*. *minor* but different time of evolution. UP-PCR generated wide range of variability among the isolates and could distinguish highly virulent and less virulent isolates. The correlation study of UP-PCR banding pattern with virulence of *S*. *sclerotiorum* identified several bands as markers of virulence. One band each of the primers 3–2, AA2M2-AS4, AA2M2-L-21, AA2M2-3-2 and L-21-3-2 were found to be significantly related to virulence of the isolates and these might be used as marker for virulence. Similar results were obtained by Sleight [30] who has identified the primers AA2M2 and 3–2 to produce the most diversified banding patterns among 11 UP-PCR primers that were used for fingerprinting of the isolates of *S*. *sclerotiorum* populations to be used as containable mycoherbicide. UP-PCR primers AS15inv and AA2M2 individually and in pair wise combination were used [31] to distinguish *Trichoderma harzianum* AS12-2 biocontrol strain from all other *Trichoderma* isolates which generated amplification products of unique size. UP-PCR revealed highest diversity among the isolates from West Bengal with 87.64% polymorphic bands, 0.369 Nei’s gene diversity and 0.531 Shannon’s diversity index. Similar findings were reported [32] with the use of 6 UP-PCR primers out of 13 to categorise 36 *Fusarium oxysporum* isolates from three cucurbit species. Likewise, UP-PCR had been applied successfully for identification of differences among populations of *Chaetomium*, *Fusarium*, *Trichoderma* and *Rhizoctonia solani* [31, 33–37]. Genetic variation in fungi may be attributed to several factors. Among these factors, mutation, population size and random genetic drift, gene flow, reproduction/mating system and selection played a vital role [38, 39]. Interplay between these factors sometimes result in either low or high variability among and within populations of these pathogens. In the present study, UP-PCR profile generated wide range of variability among *S*. *sclerotiorum* isolates, indicating usefulness of UP-PCR for assessing genetic variability among *S*. *sclerotiorum* isolates.

### Variability within and amongst the *S*. *sclerotiorum* populations

PCR with eight SSR primers generated variability among the isolates and two major groups were obtained with similarity of 70–100%. The genetic diversity of each geographical population with SSR revealed highest diversity in the Nagaland population with 61.11% polymorphic bands. The genetic distance between West Bengal and Assam population was minimum. AMOVA analysis of the banding pattern of SSR markers indicated the percentage of variation within populations to be much higher (85.07%) compared to among populations (14.93%). The results are in agreement with the findings [40] that verified high genetic diversity existed within 101 sunflower isolates of *S*. *sclerotiorum* from four different regions of China and the level of gene polymorphism was reported to be up to 85.5% within populations and only 15.5% among the populations. Earlier Atallah et al. [9] reported 92% of variability within subpopulations of 167 *S*. *sclerotiorum* isolates collected from potato fields. In Australia, Sexton et al. [41] also found 19% genetic difference within *S*. *sclerotiorum* isolates from canola fields could be accounted for variation among populations, while 79.4% genetic difference was contributed by the variation within populations.

The results of STRUCTURE analysis supported two clonal subpopulations (K = 2) for the 36 *S*. *sclerotiorum* isolates from different geographical locations with admixture and infrequent out crossing. These results were in accordance with the findings of Tok et al. [42] who studied the genetic variability of sixty *S*. *sclerotiorum* isolates of eggplant from six different geographic populations of Turkey based on MCGs, RAPD and microsatellite markers. Their results suggested that cluster groupings or genetic distance of *S*. *sclerotiorum* populations from eggplant were not distinctly related to the MCGs, geographical origin and virulence diversity. The present findings agree with Dunn et al. [43] who identified two subpopulations with STRUCTRUE for 20 isolates of *S*. *sclerotiorum* collected from 10 bean fields across New York, USA. The subpopulations were not associated with geographic location, suggesting no spatial structure to the population and predominantly clonal with limited out crossing.

## Conclusion

The present investigation reveals that the existing isolates of *S*. *sclerotiorum* in the eastern and the northeastern region of the country are admixture of two populations indicating clonal population with infrequent out crossing. Virulence of most of the isolates was of the moderate level and only few were highly virulent. UP-PCR banding pattern was correlated to the virulence variability of the isolates to certain extent. Further genetical and physiological variations will help the pathogen adapt to the varying climatic conditions and infect more new hosts. The fundamental basic research on pathobiology of *S*. *sclerotiorum* is required for understanding the basis of its wide host range and also the insights of monocotyledonous plants more resistant to *S*. *sclerotiorum* infection.

## Supporting information

S1 Table GenBank accessions of *Sclerotinia* sp. ITS sequences considered and retrieved from NCBI nucleotide database for evolutionary studies. (PDF)

S2 Table List of *S*. *sclerotiorum* isolates collected from the Northeastern states and West Bengal. (PDF)

S3 Table Virulence of the *S*. *sclerotiorum* isolates on detached fruits and potted seedlings of French bean *in vitro*. (PDF)

S4 Table Rank analysis of the *S*. *sclerotiorum* isolates based on virulence. (PDF)

S5 Table Identification of ITS sequences through BLASTN search of the GenBank database. (PDF)

S6 Table Identity matrix of *S*. *Sclerotiorum* isolates considered with other Indian and world isolates based on ITS sequence analysis. (PDF)

S7 Table Identity matrix of *S*. *sclerotiorum* isolates considered in UP-PCR analysis. (PDF)

S1 Fig Amplification of *S*. *sclerotiorum* genomic DNA with ITS primers. (PDF)

S2 Fig Phylogenetic tree of the *S*. *sclerotiorum*, *S*. *minor* and *S*. *trifoliorum* isolates. (PDF)

S3 Fig DNA amplification of *S*. *sclerotiorum* with UP-PCR primers. A: AA2M2, AS4, 3–2 and L-21 primers (top to bottom); B: AS4 & 3–2, AA2M2 & AS4, AA2M2 & 3–2, 3–2 & L-21, AA2M2 & L-21 and AS4 & L-21 primers combination.
(PDF)

S4 Fig DNA amplification of *S*. *sclerotiorum* isolates with microsatellite primers; (GA)_14,_ (CA)_9,_ (TTA)_9,_ TACA_10,_ (CT)_12,_ (CATA)_25_ and (AGAT)_14_(AAGC)_4_ primers (top to bottom). (PDF)

S1 Raw image (PDF)
