# Supplementary material for: Genetic diversity and virulence variability of Sclerotinia sclerotiorum in Eastern and Northeastern India
Source: PLoS One. 2024 Nov 25;19(11):e0312472. doi: 10.1371/journal.pone.0312472 (PMC11588274; doi:10.1371/journal.pone.0312472)
Supplement: S1 Table — (PDF) [file pone.0312472.s001.pdf]

S1 Table. GenBank accessions of the ITS sequences of *Sclerotinia* sp. considered in the current study and retrieved from NCBI nucleotide database for evolutionary studies.

Sl. No.	Accession number	Fungi	Host	Country of origin
1.	MG640571 (AS1)	*Sclerotinia sclerotiorum*	Tomato	Assam, India
2.	MG640572 (AS2)	*S. sclerotiorum*	Mustard	Assam, India
3.	MG640573 (AS3)	*S. sclerotiorum*	Marigold	Assam, India
4.	MG640586 (MZ1)	*S. sclerotiorum*	Chilli	Mizoram, India
5.	MG640587 (MZ2)	*S. sclerotiorum*	Urdbean	Mizoram, India
6.	MG640583 (NG1)	*S. sclerotiorum*	Brinjal	Nagaland, India
7.	MF563992 (NG2)	*S. sclerotiorum*	Tomato	Nagaland, India
8.	KY616637 (NG4)	*S. sclerotiorum*	Sunflower	Nagaland, India
9.	MG640588 (SK1)	*S. sclerotiorum*	Chayote	Sikkim, India
10.	MH201314(WB9)	*S. sclerotiorum*	Gerbera	West Bengal, India,
11.	MF564004(WB11)	*S. sclerotiorum*	Brinjal	West Bengal, India,
12.	MG640581(WB13)	*S. sclerotiorum*	Frenchbean	West Bengal, India,
13.	JN093300	*S. sclerotiorum*	-	Rajasthan, India
14.	KT281863	*S. sclerotiorum*	Pigeonpea	New Delhi, India
15.	JX839987	*S. sclerotiorum*	Chickpea	New Delhi, India
16.	KP676452	*S. sclerotiorum*	*Dianthus caryophyllus*	Tamil Nadu, India
17.	KF859935	*S. sclerotiorum*	-	Canada
18.	KF859934	*S. sclerotiorum*	-	Canada
19.	JQ739461	*S. sclerotiorum*	-	USA
20.	FJ810516	*S. sclerotiorum*	-	USA
21.	KX290312	*S. sclerotiorum*	-	USA
22.	KF148609	*S. sclerotiorum*	Japanese Plum, Nectarine, and Sweet Cherry	Chile
23.	KF148608	*S. sclerotiorum*	-do-	Chile
24.	JF277567	*S. sclerotiorum*	Blueberry	Argentina
25.	KR014868	*S. sclerotiorum*	-	Argentina
26.	KC848769	*S. sclerotiorum*	*Menthaspicata*	Italy
27.	AB937113	*S. sclerotiorum*	*Hibiscus rosa*	Japan
28.	AB937112	*S. sclerotiorum*	*Dahlia hortensis*	Japan
29.	HQ846942	*S. sclerotiorum*	-	China
30.	KC935388	*S. sclerotiorum*	*Arachishypogaea*	China
31.	JQ653935	*S. sclerotiorum*	*Eustoma grandiflorum*	Taiwan
32.	JQ653934	*S. sclerotiorum*	*Eustoma grandiflorum*	Taiwan
33.	KJ614565	*S. sclerotiorum*	Fennel	South Korea
34.	KJ614567	*S. sclerotiorum*	Gilliflower	South Korea
35.	JQ618848	*S. sclerotiorum*	*Euphorbia pulcherrima*	Vietnam
36.	KF791510	*S. sclerotiorum*	*Lablab purpureus*	Bangladesh

37.	KT595416	*S. sclerotiorum*	*Praecitrullusfistulosus*	Pakistan
38.	KT595415	*S. sclerotiorum*	*Trifoliumalexandrinum* (berseem)	Pakistan
39.	KU375684	*S. sclerotiorum*	*Citrus sinensis*	Iran
40.	KY049841	*S. sclerotiorum*	-	Iran
41.	KY550019	*Sclerotinia minor*	Field Aster	South Korea
42.	KM217102	*S. minor*	-	China
43.	KM217101	*S. minor*	-	China
44.	KY550016	*S. minor*	Field Aster	South Korea
45.	AY195574	*S. minor*	-	USA
46.	KP340982	*S. minor*	Sunflower	China
47.	MF964327	*S. minor*	-	USA
48.	KF859936	*Sclerotiniatrifoliorum*	-	Canada
49.	DQ904361	*S.trifoliorum*	Clover	Finland
50.	AY547267	*S.trifoliorum*	Alfalfa	China
51.	AY187080	*S.trifoliorum*	-	USA
52.	KT970794	*S.trifoliorum*	-	Pol
