# Supplementary material for: Genetic diversity and virulence variability of Sclerotinia sclerotiorum in Eastern and Northeastern India
Source: PLoS One. 2024 Nov 25;19(11):e0312472. doi: 10.1371/journal.pone.0312472 (PMC11588274; doi:10.1371/journal.pone.0312472)
Supplement: S2 Table — (PDF) [file pone.0312472.s002.pdf]

S2 Table. List of *S. sclerotiorum* isolates collected from North Eastern states and West Bengal

Sl. No	Isolate Designation	Host	Location	District	State	Geographical coordinates	NCBI Accession No.	Culture Accession No.
1.	AS1	Tomato	Rangia	Kamrup Rural	Assam	26.4373°N 91.6201˚E	MG640571	NAIMCC-F-03341
2.	AS2	Mustard	Kharupetiya	Darrang	Assam	26.4417ºN 92.0427ºE	MG640572	NAIMCC-F-03342
3.	AS3	Marigold	Mangaldai	Darrang	Assam	26.5108ºN 92.1377ºE	MG640573	NAIMCC-F-03343
4.	AS4	Frenchbean	Ramgaon	Darrang	Assam	26.5006ºN 91.8936ºE	MG640574	NAIMCC-F-03344
5.	AS5	Calendula	Mangaldai	Darrang	Assam	26.5108ºN 92.1377ºE	MG640575	NAIMCC-F-03345
6.	AS6	Sorrel	Ramgaon	Darrang	Assam	26.5006ºN 91.8936ºE	MF563990	NAIMCC-F-03346
7.	AS7	Periwinkle	Ramgaon	Darrang	Assam	26.5006ºN 91.8936ºE	MF563991	NAIMCC-F-03347
8.	AS8	Pea	Kharupetiya	Darrang	Assam	26.5096ºN 92.1352ºE	MG640576	NAIMCC-F-03348
9.	AS9	Gerbera	Kahikuchi	Kamrup Rural	Assam	25.7475ºN 92.1822ºE	MG640577	NAIMCC-F-03349
10.	MZ1	Chilli	Vengthar	Kolasib	Mizoram	24.2213ºN 92.7144ºE	MG640586	NAIMCC-F-03350
11.	MZ2	Urdbean	Chemphai	Kolasib	Mizoram	24.3430ºN 92.7494ºE	MG640587	NAIMCC-F-03365
12.	NG1	Brinjal	Domukhyia	Dimapur	Nagaland	25.9062ºN93.7275ºE	MG640583	NAIMCC-F-03351
13.	NG2	Tomato	Domukhyia	Dimapur	Nagaland	25.9062ºN93.7275ºE	MF563992	NAIMCC-F-03366
14.	NG3	Cabbage	Domukhyia	Dimapur	Nagaland	25.9062ºN93.7275ºE	MF563993	NAIMCC-F-03352
15.	NG4	Sunflower	Jharnapani	Dimapur	Nagaland	25.7558ºN 93.8438ºE	KY616637	NAIMCC-F-03353
16.	NG5	Coriander	Jharnapani	Dimapur	Nagaland	25.7573ºN 93.8330ºE	MF563994	NAIMCC-F-03364
17.	NG6	Malabar Spinach	Jharnapani	Dimapur	Nagaland	25.7573ºN 93.8330ºE	MF563995	NAIMCC-F-03367
18.	NG7	Touch me not	Jharnapani	Dimapur	Nagaland	25.7573ºN 93.8330ºE	MF563996	NAIMCC-F-03354
19.	NG8	Weed	Jharnapani	Dimapur	Nagaland	25.7573ºN 93.8330ºE	MG640584	NAIMCC-F-03355
20.	NG9	Broad leaf weed	Jharnapani	Dimapur	Nagaland	25.7573ºN 93.8330ºE	MG640585	NAIMCC-F-03356
21.	SK1	Chayote	Pabong, Namchi	South Sikkim	Sikkim	27.1772ºN88.3508º E	MG640588	NAIMCC-F-03357
22.	WB1	Capsicum	Polba	Hoogly	West Bengal	22.9594ºN 88.3069ºE	MF563997	NAIMCC-F-03358
23.	WB2	Coriander	Kanchiara	Nadia	West Bengal	23.4710ºN 88.5565ºE	MF563998	NAIMCC-F-03368
24.	WB3	Weed	Kanchiara	Nadia	West Bengal	23.4710ºN 88.5565ºE	MG640578	NAIMCC-F-03369
25.	WB4	Pea	Kanchiara	Nadia	West Bengal	23.4710ºN 88.5565ºE	MF563999	NAIMCC-F-03359
26.	WB5	Chilli	Bangaon	24PGS	West Bengal	23.0440ºN 88.8277ºE	MG640580	NAIMCC-F-03370
27.	WB6	Calendula	Mohanpur	Nadia	West Bengal	23.6565ºN 88.2254ºE	MF564000	NAIMCC-F-03371
28.	WB7	Marigold	Mohanpur	Nadia	West Bengal	23.6565ºN 88.2254ºE	MF564001	NAIMCC-F-03372
29.	WB8	Chrysanthemum	Mohanpur	Nadia	West Bengal	23.6565ºN 88.2254ºE	MG640579	NAIMCC-F-03373
30.	WB9	Gerbera	Mohanpur	Nadia	West Bengal	23.6565ºN 88.2254ºE	MH201314	NAIMCC-F-03374
31.	WB10	Dahlia	Mohanpur	Nadia	West Bengal	23.6565ºN 88.2254ºE	MF564002	NAIMCC-F-03377
32.	WB11	Brinjal	Raghunathpur	Purulia	West Bengal	23.3321ºN 86.3652ºE	MF564004	NAIMCC-F-03360
33.	WB12	Dolichosbean	Bangaon	24PGS	West Bengal	23.0440ºN 88.8277ºE	MF564003	NAIMCC-F-03375

34.	WB13	French bean	Raghunathpur	Purulia	West Bengal	23.3321ºN 86.3652ºE	MG640581	NAIMCC-F-03361
35.	WB14	Snapdragon	Mohanpur	Nadia	West Bengal	23.6565ºN 88.2254ºE	MG640582	NAIMCC-F-03362
36.	WB15	Daisy	Mohanpur	Nadia	West Bengal	23.6565ºN 88.2254ºE	MH201315	NAIMCC-F-03376
