# Supplementary material for: Genetic diversity and virulence variability of Sclerotinia sclerotiorum in Eastern and Northeastern India
Source: PLoS One. 2024 Nov 25;19(11):e0312472. doi: 10.1371/journal.pone.0312472 (PMC11588274; doi:10.1371/journal.pone.0312472)
Supplement: S3 Table — (PDF) [file pone.0312472.s003.pdf]

S3 Table. Virulence of the *S. sclerotiorum* isolates on detached fruits and potted seedlings of French bean *in vitro*

**Sl.**	**Isolate**	**AvLL**			**Virulence**	**AULPC**			**Virulence**	**Grading**			**Virulence**	**AUDPC**			**Virulence**
		R1	R2	R3		R1	R2	R3		R1	R2	R3		R1	R2	R3	
1	AS1	7.3	6.8	11.3	MV	11.75	10	18.25	MV	8	8	9	HV	48	48	52	MV
2	AS2	7.5	4.8	6.5	MV	10.35	9.1	9.95	LV	8	9	9	HV	48	52	52	MV
3	AS3	4	5.2	5	LV	4.7	6.9	6.6	LV	5	4	4	MV	26	28	30	LV
4	AS4	11.5	10	11.7	HV	18.6	19.95	23.25	HV	9	9	9	HV	68	66	60	HV
5	AS5	8.7	8.2	7.4	MV	11.75	10.1	10.6	MV	8	8	9	HV	42	44	50	MV
6	AS6	13.3	8.5	10.9	HV	19.75	16.25	20.35	MV	9	9	9	HV	60	66	62	HV
7	AS7	7.5	9	7.7	MV	10.05	12.7	10.65	MV	8	9	9	HV	46	46	52	MV
8	AS8	7.5	7	6.8	MV	9.15	9	9.4	LV	7	8	8	HV	40	46	52	MV
9	AS9	9	6.5	6	MV	12	12	12.25	MV	7	7	8	HV	42	44	50	MV
10	MZ1	4	1.5	3.5	LV	6	0.75	5.95	LV	4	5	6	MV	30	30	38	MV
11	MZ2	7	5	6	MV	10	7.3	10.05	LV	7	7	8	HV	42	48	54	MV
12	NG1	8	9	9.3	MV	13	13.5	15.9	MV	8	9	8	HV	44	54	54	MV
13	NG2	8.7	6.6	6.3	MV	12.55	7.1	7.65	LV	7	8	9	HV	36	36	42	MV
14	NG3	7	4.5	6	MV	9.9	6.75	8.5	LV	6	6	7	HV	34	38	44	MV
15	NG4	9.5	9	9	MV	15.5	16	13.15	MV	9	9	9	HV	56	58	62	MV
16	NG5	4.5	4.3	3	LV	5.25	6.55	5.5	LV	5	4	4	MV	32	28	26	LV
17	NG6	8	7	7.5	MV	11	11.25	12.5	MV	7	8	9	HV	40	46	50	MV
18	NG7	4.5	7	6	MV	7.75	11	11.5	MV	7	8	7	HV	42	46	50	MV
19	NG8	7.5	5.5	6	MV	9.75	7.75	8	LV	6	5	6	MV	34	26	34	MV
20	NG9	2.5	4	5	LV	4.75	5.8	8	LV	5	6	6	MV	32	38	40	MV
21	SK1	9	7.5	9.5	MV	13.25	12.4	14.25	MV	9	8	9	HV	50	58	62	MV
22	WB1	11.5	12	11	HV	20.5	21	19.25	HV	9	9	9	HV	62	66	64	HV
23	WB2	3.5	4	5.5	LV	3.45	4.9	6.25	LV	5	5	6	MV	26	30	30	LV
24	WB3	6	5	8.5	MV	9.8	6.3	16.3	MV	7	7	8	HV	42	54	52	MV
25	WB4	5	5	4	LV	6	7	5	LV	5	6	7	MV	30	28	32	LV
26	WB5	5.5	4	6.5	MV	7.45	5.5	7.25	LV	6	7	7	HV	32	34	36	MV
27	WB6	5	6	6.5	MV	8.5	10.75	9	LV	6	7	8	HV	34	40	36	MV
28	WB7	11.5	11	12	HV	16.55	16.8	18.25	MV	9	9	9	HV	52	64	62	MV
29	WB8	4.5	3	5	LV	6.75	6.4	7	LV	5	4	3	MV	32	26	26	LV
30	WB9	7	5	8	MV	7.5	7.5	9.2	LV	7	8	8	HV	36	40	46	MV
31	WB10	6	4	4.5	LV	7.5	6.1	7.15	LV	5	7	7	HV	32	32	34	MV
32	WB11	7.5	6	8	MV	10.15	10.05	11.5	MV	7	7	8	HV	40	42	50	MV
33	WB12	8	7.5	6	MV	12.25	10.25	8.5	MV	7	8	8	HV	36	46	48	MV
34	WB13	7	8	7.5	MV	12.05	12.75	11.05	MV	7	8	9	HV	42	56	58	MV

35	WB14	5.5	5	6	MV	6.65	7.5	8.2	LV	6	6	5	MV	34	36	36	MV
36	WB15	6.5	6	5	MV	8.15	7	6.6	LV	6	7	8	HV	34	42	48	MV
SEM (±)		0.62				0.98				0.338				1.95			
CD		1.74				2.77				0.952				5.5			
