# Supplementary material for: Genetic diversity and virulence variability of Sclerotinia sclerotiorum in Eastern and Northeastern India
Source: PLoS One. 2024 Nov 25;19(11):e0312472. doi: 10.1371/journal.pone.0312472 (PMC11588274; doi:10.1371/journal.pone.0312472)
Supplement: S4 Table — (PDF) [file pone.0312472.s004.pdf]

S4 Table. Rank analysis of the *S. sclerotiorum* isolates based on virulence

Sl.	Isolate	Lesion length	AULPC	Grading	AUDPC	Cumulative ranking	Ranking of the cumulative ranking
1	AS1	8	7	9	10	34	29
2	AS2	21	18	6	8	53	24
3	AS3	30	32	34	35	131	5
4	AS4	3	1	1	1	6	35
5	AS5	9	13	9	16	47	26
6	AS6	4	3	1	3	11	33
7	AS7	10	12	6	12	40	27
8	AS8	17	20	15	14	66	19
9	AS9	14	9	18	16	57	23
10	MZ1	36	36	33	29	134	2
11	MZ2	22	21	18	12	73	16
12	NG1	6	6	9	8	29	30
13	NG2	13	22	12	24	71	18
14	NG3	23	24	26	23	96	12
15	NG4	5	5	1	5	16	32
16	NG5	34	34	34	33	135	1
17	NG6	11	11	12	16	50	25
18	NG7	23	17	18	14	72	17
19	NG8	20	23	29	31	103	11
20	NG9	35	31	29	25	120	7
21	SK1	7	8	6	6	27	31
22	WB1	1	2	1	2	6	35
23	WB2	32	35	32	33	132	4
24	WB3	19	14	18	10	61	22

25	WB4	31	33	28	32	124	6
26	WB5	28	29	25	28	110	9
27	WB6	23	19	23	25	90	14
28	WB7	1	4	1	4	10	34
29	WB8	33	30	36	35	134	2
30	WB9	18	25	15	22	80	15
31	WB10	29	28	26	29	112	8
32	WB11	14	15	18	19	66	19
33	WB12	14	16	15	20	65	21
34	WB13	11	10	12	7	40	27
35	WB14	27	26	29	27	109	10
36	WB15	23	27	23	21	94	13
