# Supplementary material for: Genetic diversity and virulence variability of Sclerotinia sclerotiorum in Eastern and Northeastern India
Source: PLoS One. 2024 Nov 25;19(11):e0312472. doi: 10.1371/journal.pone.0312472 (PMC11588274; doi:10.1371/journal.pone.0312472)
Supplement: S5 Table — (PDF) [file pone.0312472.s005.pdf]

S5 Table. Identification of ITS sequences through BLASTN search of the GenBank database

Sl. No	Isolate No.	Accession No.	Max Identity	BLAST Seq. ID	Identified as
1.	AS1	MG640571	100%	MH539643.1	*Sclerotinia sclerotiorum*
2.	AS2	MG640572	100%	MH457168.1	*S. sclerotiorum*
3.	AS3	MG640573	100%	MG640587.1	*S. sclerotiorum*
4.	AS4	MG640574	100%	MH393290.1	*S. sclerotiorum*
5.	AS5	MG640575	100%	MH393289.1	*S. sclerotiorum*
6.	AS6	MF563990	100%	MH298773.1	*S. sclerotiorum*
7.	AS7	MF563991	100%	MH327997.1	*S. sclerotiorum*
8.	AS8	MG640576	100%	KY947523.1	*S. sclerotiorum*
9.	AS9	MG640577	100%	MH298770.1	*S. sclerotiorum*
10.	MZ1	MG640586	100%	MH298773.1	*S. sclerotiorum*
11.	MZ2	MG640587	100%	CP017820.1	*S. sclerotiorum*
12.	NG1	MG640583	100%	KY848798.1	*S. sclerotiorum*
13.	NG2	MF563992	100%	KY947523.1	*S. sclerotiorum*
14.	NG3	MF563993	100%	KY848795.1	*S. sclerotiorum*
15.	NG4	KY616637	100%	KX427540.1	*S. sclerotiorum*
16.	NG5	MF563994	100%	MH298771.1	*S. sclerotiorum*
17.	NG6	MF563995	100%	MH327997.1	*S. sclerotiorum*
18.	NG7	MF563996	100%	MH298768.1	*S. sclerotiorum*
19.	NG8	MG640584	100%	MH298772.1	*S. sclerotiorum*
20.	NG9	MG640585	100%	MH298769.1	*S. sclerotiorum*
21.	SK1	MG640588	100%	MG516699.1	*S. sclerotiorum*
22.	WB1	MF563997	100%	MG516701.1	*S. sclerotiorum*
23.	WB2	MF563998	100%	LC271233.1	*S. sclerotiorum*
24.	WB3	MG640578	100%	KX184720.1	*S. sclerotiorum*
25.	WB4	MF563999	100%	KY848793.1	*S. sclerotiorum*
26.	WB5	MG640580	99%	KY848790.1	*S. sclerotiorum*
27.	WB6	MF564000	100%	MG249967.1	*S. sclerotiorum*
28.	WB7	MF564001	100%	LC318723.1	*S. sclerotiorum*
29.	WB8	MG640579	100%	MG516708.1	*S. sclerotiorum*
30.	WB9	MH201314	100%	JN012605.1	*S. sclerotiorum*
31.	WB10	MF564002	100%	KY947523.1	*S. sclerotiorum*
32.	WB11	MF564004	100%	MH298773.1	*S. sclerotiorum*
33.	WB12	MF564003	99%	MH539643.1	*S. sclerotiorum*
34.	WB13	MG640581	100%	MH539643.1	*S. sclerotiorum*
35.	WB14	MG640582	99%	KM272357.1	*S. sclerotiorum*
36.	WB15	MH201315	100%	MH393290.1	*S. sclerotiorum*
