# Supplementary material for: Genetic diversity and virulence variability of Sclerotinia sclerotiorum in Eastern and Northeastern India
Source: PLoS One. 2024 Nov 25;19(11):e0312472. doi: 10.1371/journal.pone.0312472 (PMC11588274; doi:10.1371/journal.pone.0312472)
Supplement: S6 Table — (PDF) [file pone.0312472.s006.pdf]

S6 Table. Identity matrix of *S. Sclerotiorum* isolates considered with other Indian and world isolates based on ITS sequence analysis

	AS1	AS2	AS3	MZ1	MZ2	NG1	NG2	NG4	SK1	WB9	WB11	WB13	JN093300_Rajasthan	KT281863_New_Delhi	JX839987_New_Delhi	KP676452_Tamil_Nadu	KF859935_Canada	KF859934_Canada	JQ739461_USA	FJ810516_USA	KX290312_USA	KF148609_Chile	KF148608_Chile	JF277567_Argentina	KR014868_Argentina	KC848769_Italy	AB937113_Japan	AB937112_Japan	HQ846942_China	KC935388_China	JQ653935_Taiwan	JQ653934_Taiwan	KJ614565_S._Korea	KJ614567_S._Korea	JQ618848_Vietnam	KF791510_Bangladesh	KT595416_Pakistan	KT595415_Pakistan	KU375684_Iran	KY049841_Iran	KY550019_Sm_S.Korea	KM217102_Sm_China	KM217101_Sm_China	KY550016_Sm_S.Korea	AY195574_Sm_USA	KP340982_Sm_China	MF964327_Sm_USA	KF859936_St_Canada	DQ904361_St_Finland	AY547267_St_China	AY187080_St_USA	KT970794_St_Poland
AS1	ID																																																			
AS2	100	ID																																																		
AS3	100	100	ID																																																	
MZ1	100	100	100	ID																																																
MZ2	100	100	100	100	ID																																															
NG1	100	100	100	100	100	ID																																														
NG2	100	100	100	100	100	100	ID																																													
NG4	100	100	100	100	100	100	100	ID																																												
SK1	100	100	100	100	100	100	100	100	ID																																											
WB9	100	100	100	100	100	100	100	100	100	ID																																										
WB11	100	100	100	100	100	100	100	100	100	100	ID																																									
WB13	100	100	100	100	100	100	100	100	100	100	100	ID																																								
JN093300_Rajasthan	99.5	99.5	99.5	99.5	99.5	99.5	99.5	99.5	99.5	99.5	99.5	99.5	ID																																							
KT281863_New_Delhi	100	100	100	100	100	100	100	100	100	100	100	100	99.5	ID																																						
JX839987_New_Delhi	99.2	99.2	99.2	99.2	99.2	99.2	99.2	99.2	99.2	99.2	99.2	99.2	98.7	99.2	ID																																					
KP676452_Tamil_Nadu	99.5	99.5	99.5	99.5	99.5	99.5	99.5	99.5	99.5	99.5	99.5	99.5	99	99.5	98.7	ID																																				
KF859935_Canada	97.3	97.3	97.3	97.3	97.3	97.3	97.3	97.3	97.3	97.3	97.3	97.3	96.8	97.3	96.5	96.8	ID																																			
KF859934_Canada	97.5	97.5	97.5	97.5	97.5	97.5	97.5	97.5	97.5	97.5	97.5	97.5	97	97.5	96.8	97	99.7	ID																																		
JQ739461_USA	100	100	100	100	100	100	100	100	100	100	100	100	99.5	100	99.2	99.5	97.3	97.5	ID																																	
FJ810516_USA	100	100	100	100	100	100	100	100	100	100	100	100	99.5	100	99.2	99.5	97.3	97.5	100	ID																																
KX290312_USA	100	100	100	100	100	100	100	100	100	100	100	100	99.5	100	99.2	99.5	97.3	97.5	100	100	ID																															
KF148609_Chile	100	100	100	100	100	100	100	100	100	100	100	100	99.5	100	99.2	99.5	97.3	97.5	100	100	100	ID																														
KF148608_Chile	100	100	100	100	100	100	100	100	100	100	100	100	99.5	100	99.2	99.5	97.3	97.5	100	100	100	100	ID																													
JF277567_Argentina	100	100	100	100	100	100	100	100	100	100	100	100	99.5	100	99.2	99.5	97.3	97.5	100	100	100	100	100	ID																												
KR014868_Argentina	98.5	98.5	98.5	98.5	98.5	98.5	98.5	98.5	98.5	98.5	98.5	98.5	98	98.5	97.8	98	95.8	96.1	98.5	98.5	98.5	98.5	98.5	98.5	ID																											
KC848769_Italy	100	100	100	100	100	100	100	100	100	100	100	100	99.5	100	99.2	99.5	97.3	97.5	100	100	100	100	100	100	98.5	ID																										
AB937113_Japan	100	100	100	100	100	100	100	100	100	100	100	100	99.5	100	99.2	99.5	97.3	97.5	100	100	100	100	100	100	98.5	100	ID																									
AB937112_Japan	100	100	100	100	100	100	100	100	100	100	100	100	99.5	100	99.2	99.5	97.3	97.5	100	100	100	100	100	100	98.5	100	100	ID																								
HQ846942_China	100	100	100	100	100	100	100	100	100	100	100	100	99.5	100	99.2	99.5	97.3	97.5	100	100	100	100	100	100	98.5	100	100	100	ID																							
KC935388_China	100	100	100	100	100	100	100	100	100	100	100	100	99.5	100	99.2	99.5	97.3	97.5	100	100	100	100	100	100	98.5	100	100	100	100	ID																						
JQ653935_Taiwan	100	100	100	100	100	100	100	100	100	100	100	100	99.5	100	99.2	99.5	97.3	97.5	100	100	100	100	100	100	98.5	100	100	100	100	100	ID																					
JQ653934_Taiwan	100	100	100	100	100	100	100	100	100	100	100	100	99.5	100	99.2	99.5	97.3	97.5	100	100	100	100	100	100	98.5	100	100	100	100	100	100	ID																				
KJ614565_S._Korea	100	100	100	100	100	100	100	100	100	100	100	100	99.5	100	99.2	99.5	97.3	97.5	100	100	100	100	100	100	98.5	100	100	100	100	100	100	100	ID																			
KJ614567_S._Korea	100	100	100	100	100	100	100	100	100	100	100	100	99.5	100	99.2	99.5	97.3	97.5	100	100	100	100	100	100	98.5	100	100	100	100	100	100	100	100	ID																		
JQ618848_Vietnam	100	100	100	100	100	100	100	100	100	100	100	100	99.5	100	99.2	99.5	97.3	97.5	100	100	100	100	100	100	98.5	100	100	100	100	100	100	100	100	100	ID																	
KF791510_Bangladesh	100	100	100	100	100	100	100	100	100	100	100	100	99.5	100	99.2	99.5	97.3	97.5	100	100	100	100	100	100	98.5	100	100	100	100	100	100	100	100	100	100	ID																
KT595416_Pakistan	99.5	99.5	99.5	99.5	99.5	99.5	99.5	99.5	99.5	99.5	99.5	99.5	99.5	99.5	98.7	99	96.8	97	99.5	99.5	99.5	99.5	99.5	99.5	98	99.5	99.5	99.5	99.5	99.5	99.5	99.5	99.5	99.5	99.5	99.5	ID															
KT595415_Pakistan	99.2	99.2	99.2	99.2	99.2	99.2	99.2	99.2	99.2	99.2	99.2	99.2	98.7	99.2	98.5	98.7	96.8	97	99.2	99.2	99.2	99.2	99.2	99.2	97.8	99.2	99.2	99.2	99.2	99.2	99.2	99.2	99.2	99.2	99.2	99.2	98.7	ID														
KU375684_Iran	100	100	100	100	100	100	100	100	100	100	100	100	99.5	100	99.2	99.5	97.3	97.5	100	100	100	100	100	100	98.5	100	100	100	100	100	100	100	100	100	100	100	99.5	99.2	ID													
KY049841_Iran	99	99	99	99	99	99	99	99	99	99	99	99	98.5	99	98.3	98.5	96.3	96.6	99	99	99	99	99	99	97.5	99	99	99	99	99	99	99	99	99	99	99	99	98.3	99	ID												
KY550019_Sm_S.Korea	99.2	99.2	99.2	99.2	99.2	99.2	99.2	99.2	99.2	99.2	99.2	99.2	98.7	99.2	98.5	98.7	96.5	96.8	99.2	99.2	99.2	99.2	99.2	99.2	98	99.2	99.2	99.2	99.2	99.2	99.2	99.2	99.2	99.2	99.2	99.2	98.7	98.5	99.2	98.3	ID											
KM217102_Sm_China	99.2	99.2	99.2	99.2	99.2	99.2	99.2	99.2	99.2	99.2	99.2	99.2	98.7	99.2	98.5	98.7	96.5	96.8	99.2	99.2	99.2	99.2	99.2	99.2	98	99.2	99.2	99.2	99.2	99.2	99.2	99.2	99.2	99.2	99.2	99.2	98.7	98.5	99.2	98.3	100	ID										
KM217101_Sm_China	99.2	99.2	99.2	99.2	99.2	99.2	99.2	99.2	99.2	99.2	99.2	99.2	98.7	99.2	98.5	98.7	96.5	96.8	99.2	99.2	99.2	99.2	99.2	99.2	98	99.2	99.2	99.2	99.2	99.2	99.2	99.2	99.2	99.2	99.2	99.2	98.7	98.5	99.2	98.3	100	100	ID									
KY550016_Sm_S.Korea	99.2	99.2	99.2	99.2	99.2	99.2	99.2	99.2	99.2	99.2	99.2	99.2	98.7	99.2	98.5	98.7	96.5	96.8	99.2	99.2	99.2	99.2	99.2	99.2	98	99.2	99.2	99.2	99.2	99.2	99.2	99.2	99.2	99.2	99.2	99.2	98.7	98.5	99.2	98.3	100	100	100	ID								
AY195574_Sm_USA	99.2	99.2	99.2	99.2	99.2	99.2	99.2	99.2	99.2	99.2	99.2	99.2	98.7	99.2	98.5	98.7	96.5	96.8	99.2	99.2	99.2	99.2	99.2	99.2	98	99.2	99.2	99.2	99.2	99.2	99.2	99.2	99.2	99.2	99.2	99.2	98.7	98.5	99.2	98.3	100	100	100	100	ID							
KP340982_Sm_China	99.2	99.2	99.2	99.2	99.2	99.2	99.2	99.2	99.2	99.2	99.2	99.2	98.7	99.2	98.5	98.7	96.5	96.8	99.2	99.2	99.2	99.2	99.2	99.2	98	99.2	99.2	99.2	99.2	99.2	99.2	99.2	99.2	99.2	99.2	99.2	98.7	98.5	99.2	98.3	100	100	100	100	100	ID						
MF964327_Sm_USA	99.2	99.2	99.2	99.2	99.2	99.2	99.2	99.2	99.2	99.2	99.2	99.2	98.7	99.2	98.5	98.7	96.5	96.8	99.2	99.2	99.2	99.2	99.2	99.2	98	99.2	99.2	99.2	99.2	99.2	99.2	99.2	99.2	99.2	99.2	99.2	98.7	98.5	99.2	98.3	100	100	100	100	100	100	ID					
KF859936_St_Canada	99.7	99.7	99.7	99.7	99.7	99.7	99.7	99.7	99.7	99.7	99.7	99.7	99.2	99.7	99	99.2	97	97.3	99.7	99.7	99.7	99.7	99.7	99.7	98.2	99.7	99.7	99.7	99.7	99.7	99.7	99.7	99.7	99.7	99.7	99.7	99.2	99	99.7	98.7	99	99	99	99	99	99	99	ID				
DQ904361_St_Finland	96.8	96.8	96.8	96.8	96.8	96.8	96.8	96.8	96.8	96.8	96.8	96.8	96.3	96.8	96	96.3	94.1	94.4	96.8	96.8	96.8	96.8	96.8	96.8	95.3	96.8	96.8	96.8	96.8	96.8	96.8	96.8	96.8	96.8	96.8	96.8	96.3	96	96.8	95.8	96	96	96	96	96	96	96	97	ID			
AY547267_St_China	99.5	99.5	99.5	99.5	99.5	99.5	99.5	99.5	99.5	99.5	99.5	99.5	99	99.5	98.7	99	96.8	97	99.5	99.5	99.5	99.5	99.5	99.5	98	99.5	99.5	99.5	99.5	99.5	99.5	99.5	99.5	99.5	99.5	99.5	99	98.7	99.5	98.5	98.7	98.7	98.7	98.7	98.7	98.7	98.7	99.7	97	ID		
AY187080_St_USA	99.5	99.5	99.5	99.5	99.5	99.5	99.5	99.5	99.5	99.5	99.5	99.5	99	99.5	98.7	99	97.3	97.5	99.5	99.5	99.5	99.5	99.5	99.5	98	99.5	99.5	99.5	99.5	99.5	99.5	99.5	99.5	99.5	99.5	99.5	99	98.7	99.5	98.5	98.7	98.7	98.7	98.7	98.7	98.7	98.7	99.7	96.8	99.5	ID	
KT970794_St_Poland	98.2	98.2	98.2	98.2	98.2	98.2	98.2	98.2	98.2	98.2	98.2	98.2	97.8	98.2	98	97.8	95.6	95.8	98.2	98.2	98.2	98.2	98.2	98.2	96.8	98.2	98.2	98.2	98.2	98.2	98.2	98.2	98.2	98.2	98.2	98.2	97.8	97.5	98.2	97.3	97.5	97.5	97.5	97.5	97.5	97.5	97.5	98.5	95.8	98.7	98.2	ID
