# Supplementary material for: Genetic diversity and virulence variability of Sclerotinia sclerotiorum in Eastern and Northeastern India
Source: PLoS One. 2024 Nov 25;19(11):e0312472. doi: 10.1371/journal.pone.0312472 (PMC11588274; doi:10.1371/journal.pone.0312472)
Supplement: S7 Table — (PDF) [file pone.0312472.s007.pdf]

S7 Table. Identity matrix of *S. Sclerotiorum* isolates considered on UP-PCR analysis

	WB1	WB2	WB3	WB4	WB5	WB6	WB7	WB8	WB9	WB10	WB11	WB12	WB13	WB14	WB15	AS1	AS2	AS3	AS4	AS5	AS6	AS7	AS8	AS9	NG1	NG2	NG3	NG4	NG5	NG6	NG7	NG8	NG9	MZ1	MZ2	SK1
WB1	ID																																			
WB2	64.38	ID																																		
WB3	14.58	10.96	ID																																	
WB4	60	78.08	14.04	ID																																
WB5	52.24	70.67	10.53	75	ID																															
WB6	10.42	8.22	7.69	8.62	10.91	ID																														
WB7	58.57	82.67	10.77	86.15	83.08	9.38	ID																													
WB8	9.62	13.7	5.88	15.52	18.18	6.67	13.85	ID																												
WB9	16.33	13.7	0	17.54	18.18	0	15.63	0	ID																											
WB10	46.43	44	10.26	53.33	55.17	13.89	54.69	7.14	28.57	ID																										
WB11	47.76	66.67	11.11	65.15	57.35	7.41	61.11	16.98	8.77	35.94	ID																									
WB12	50.88	51.35	14.63	52.38	40.3	15.38	47.14	6.52	11.36	48	56.9	ID																								
WB13	57.38	62.67	16.33	65.63	55.22	5.77	61.43	18	13.46	42.37	62.9	49.15	ID																							
WB14	38	30.14	7.14	36.21	35.09	7.69	34.38	14.29	14.29	32.56	29.82	24.49	33.96	ID																						
WB15	21.15	20.27	33.33	25.86	18.33	4.76	21.21	18.18	0	15.91	25.93	25	27.45	26.67	ID																					
AS1	24.49	19.18	15.79	24.56	15	11.11	20	9.09	33.33	22.5	20	23.26	26	28.57	20	ID																				
AS2	10.64	6.85	8.33	8.77	9.09	22.22	7.81	15.38	7.14	14.29	7.55	12.82	10.2	8	10.53	18.75	ID																			
AS3	47.62	56.58	5.88	49.28	46.38	6.12	52.78	14.29	12	44.64	53.13	51.79	55.74	30.77	16.98	22.45	8.51	ID																		
AS4	44.07	48	15	55.74	55	15.79	52.24	11.63	20	46	38.46	42.59	42.62	27.66	20	20.93	10.26	42.37	ID																	
AS5	50	64.47	11.11	67.69	64.62	11.54	65.71	16.98	19.23	55.36	55.22	54.24	50.75	29.82	21.43	22.22	9.62	53.13	66.67	ID																
AS6	48.48	58.97	13.46	63.64	53.62	11.76	59.72	8.93	17.31	50.88	45.07	57.89	47.06	25.86	21.82	20.37	9.8	51.56	67.92	74.58	ID															
AS7	50	58.44	14	58.21	50.72	12.24	56.94	11.32	11.32	44.83	53.03	49.15	53.13	29.09	25	23.53	10.2	46.15	47.46	60.32	63.93	ID														
AS8	2.08	1.35	11.11	1.72	1.79	14.29	1.54	9.09	0	2.78	1.89	2.5	2	4.35	5.88	6.67	16.67	2.13	2.56	1.89	1.92	2	ID													
AS9	12	12.33	6.25	13.79	14.29	7.14	14.06	26.67	18.75	12.82	12.96	6.67	13.73	24	8.7	21.05	7.69	14.58	9.3	12.96	9.09	16	10	ID												
NG1	2.13	1.37	12.5	1.75	1.82	16.67	1.56	10	0	2.86	1.92	2.56	2.04	4.55	6.25	7.14	20	2.17	2.63	1.92	1.96	2.04	50	11.11	ID											
NG2	18.03	27.27	3.13	30.16	29.03	14.81	28.99	12.9	16.67	27.66	26.23	33.33	19.35	20.51	10.81	21.88	11.11	31.48	36.96	37.5	43.4	23.33	8	17.24	4	ID										
NG3	32.69	25	15.38	27.42	28.33	16.67	28.36	6.67	6.67	29.55	17.46	32.61	29.09	15.79	18.75	12.5	17.39	21.43	39.53	32.14	35.19	31.48	4.35	0	0	27.03	ID									
NG4	4.26	2.74	25	3.51	3.64	0	3.13	0	0	5.71	1.89	5.13	4.08	4.35	5.88	0	0	2.13	5.26	3.85	3.92	4.08	0	0	0	0	9.09	ID								
NG5	11.11	14.67	0	14.75	17.24	11.76	16.67	15	9.52	14.29	12.07	15.56	10.71	9.38	7.41	3.85	5.88	9.26	24.39	20.37	18.52	12.73	0	4.76	0	26.67	40	0	ID							
NG6	44.62	50	3.77	48.57	41.67	10.42	48	11.76	14	43.86	39.44	50.88	35.21	25.45	16.67	19.61	10.64	52.46	46.55	57.14	66.1	47.69	2.08	9.8	2.13	44	30.19	0	22.45	ID						
NG7	48.44	55.13	12	52.17	49.28	10.2	51.35	11.54	13.73	40.68	40.85	52.63	40.58	29.63	23.08	16.98	10.42	46.88	56.36	58.73	67.8	51.56	2.04	9.62	2.08	40.38	40	4.17	22	75.93	ID					
NG8	15.69	13.33	0	11.29	11.67	5.88	11.76	4.76	15.79	17.5	8.47	10.87	8.93	17.24	3.7	18.18	6.25	16	16.28	14.29	14.55	15.09	0	16.67	0	19.35	13.33	0	8.7	20.41	20	ID				
NG9	26	21.92	4.35	25.86	26.79	10	25	13.04	23.81	27.5	23.64	22.22	27.45	40.74	18.52	30.43	16.67	21.57	22.73	23.64	19.64	27.45	5.88	31.58	6.25	32.26	22.58	0	26.09	26	25.49	16.67	ID			
MZ1	27.45	22.97	4	22.95	25.86	9.09	22.39	21.74	7.69	20.45	25	11.76	26.42	37.93	13.33	18.52	9.52	25.49	19.15	20.69	15	26.42	5.26	35	5.56	13.16	11.11	0	10.71	22.64	20	25	41.67	ID		
MZ2	31.15	43.24	24.24	45.16	49.15	2.63	42.65	22.86	16.22	25.93	44.07	30.91	41.38	27.91	28.95	23.68	5.56	36.21	36.54	46.55	40	41.38	2.94	23.53	3.03	38.1	17.02	6.06	12.2	31.15	42.11	15.38	32.43	24.39	ID	
SK1	12.24	10.96	6.67	12.07	12.5	16.67	12.5	12.5	20	19.44	9.09	11.9	11.76	20	4.35	22.22	18.18	12.5	15	13.21	13.46	14	11.11	41.67	12.5	22.22	11.11	0	10.53	14.58	14.29	33.33	33.33	23.81	13.89	ID
