# Supplementary figures and images for: Genetic diversity and virulence variability of Sclerotinia sclerotiorum in Eastern and Northeastern India
Source: PLoS One. 2024 Nov 25;19(11):e0312472. doi: 10.1371/journal.pone.0312472 (PMC11588274; doi:10.1371/journal.pone.0312472)

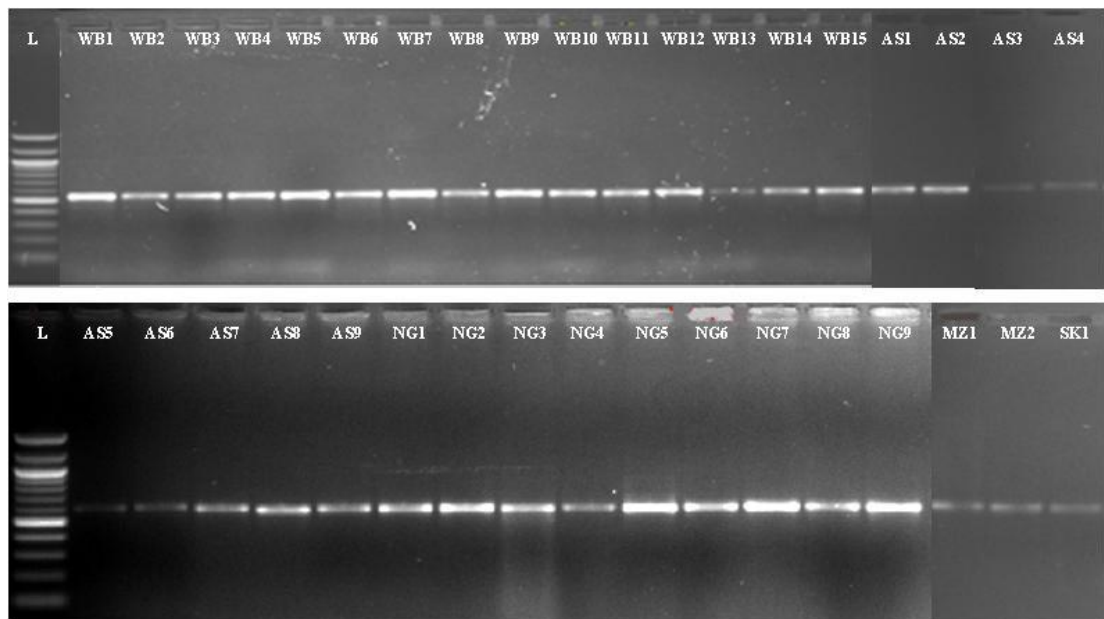

**S1 Fig. Amplification of *S. sclerotiorum* genomic DNA with ITS primers**

Supplement: S1 Fig — (PDF) [file pone.0312472.s008.pdf]

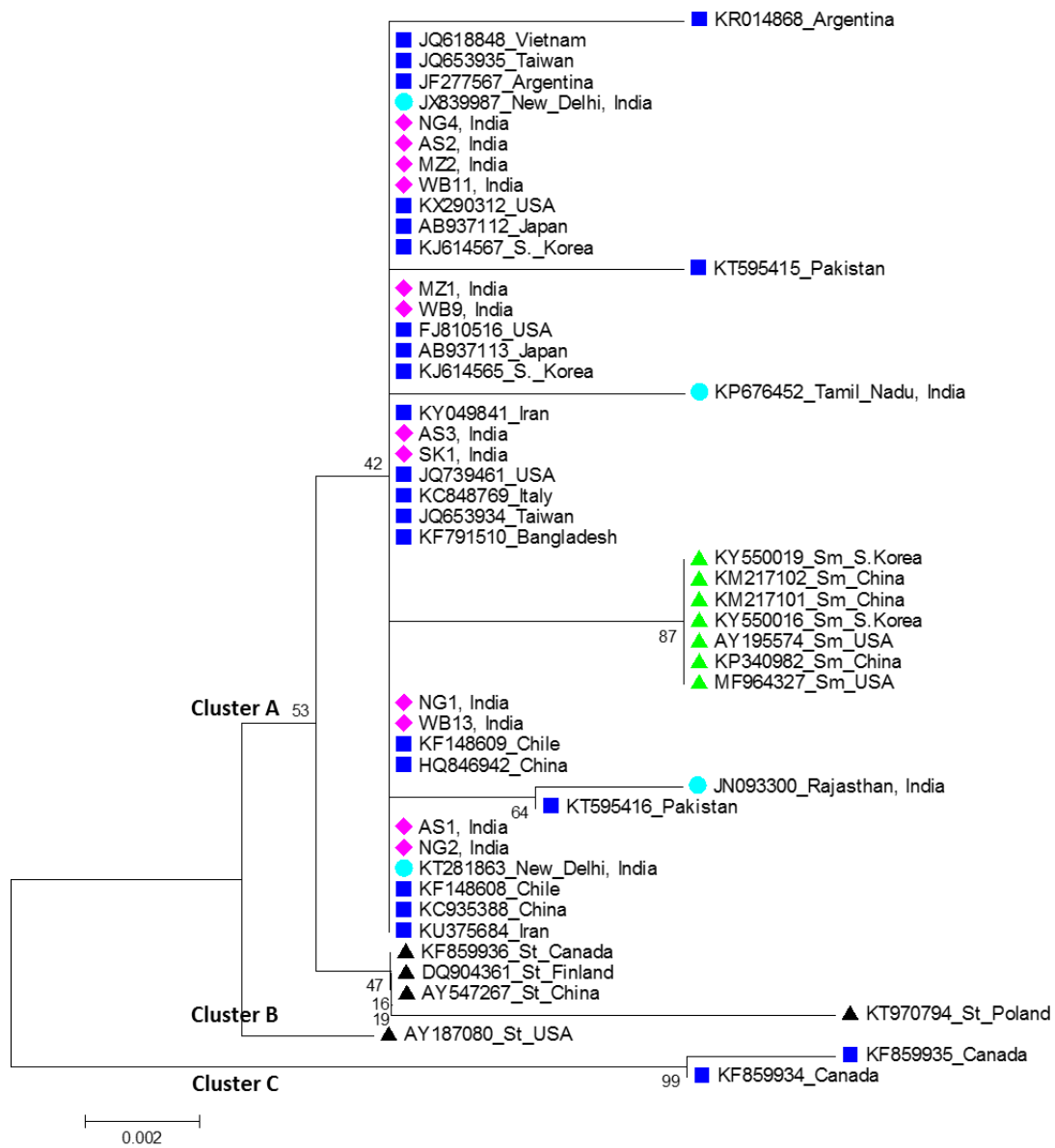

**S2 Fig.** Phylogenetic tree of the *S. sclerotiorum*, *S. minor* and *S. trifoliorum* isolates

Supplement: S2 Fig — (PDF) [file pone.0312472.s009.pdf]
