# Supplementary material for: Genetic diversity and virulence variability of Sclerotinia sclerotiorum in Eastern and Northeastern India
Source: PLoS One. 2024 Nov 25;19(11):e0312472. doi: 10.1371/journal.pone.0312472 (PMC11588274; doi:10.1371/journal.pone.0312472)
Supplement: S3 Fig — A: AA2M2, AS4, 3–2 and L-21 primers (top to bottom); B: AS4 & 3–2, AA2M2 & AS4, AA2M2 & 3–2, 3–2 & L-21, AA2M2 & L-21 and AS4 & L-21 primers combination. (PDF) [file pone.0312472.s010.pdf]

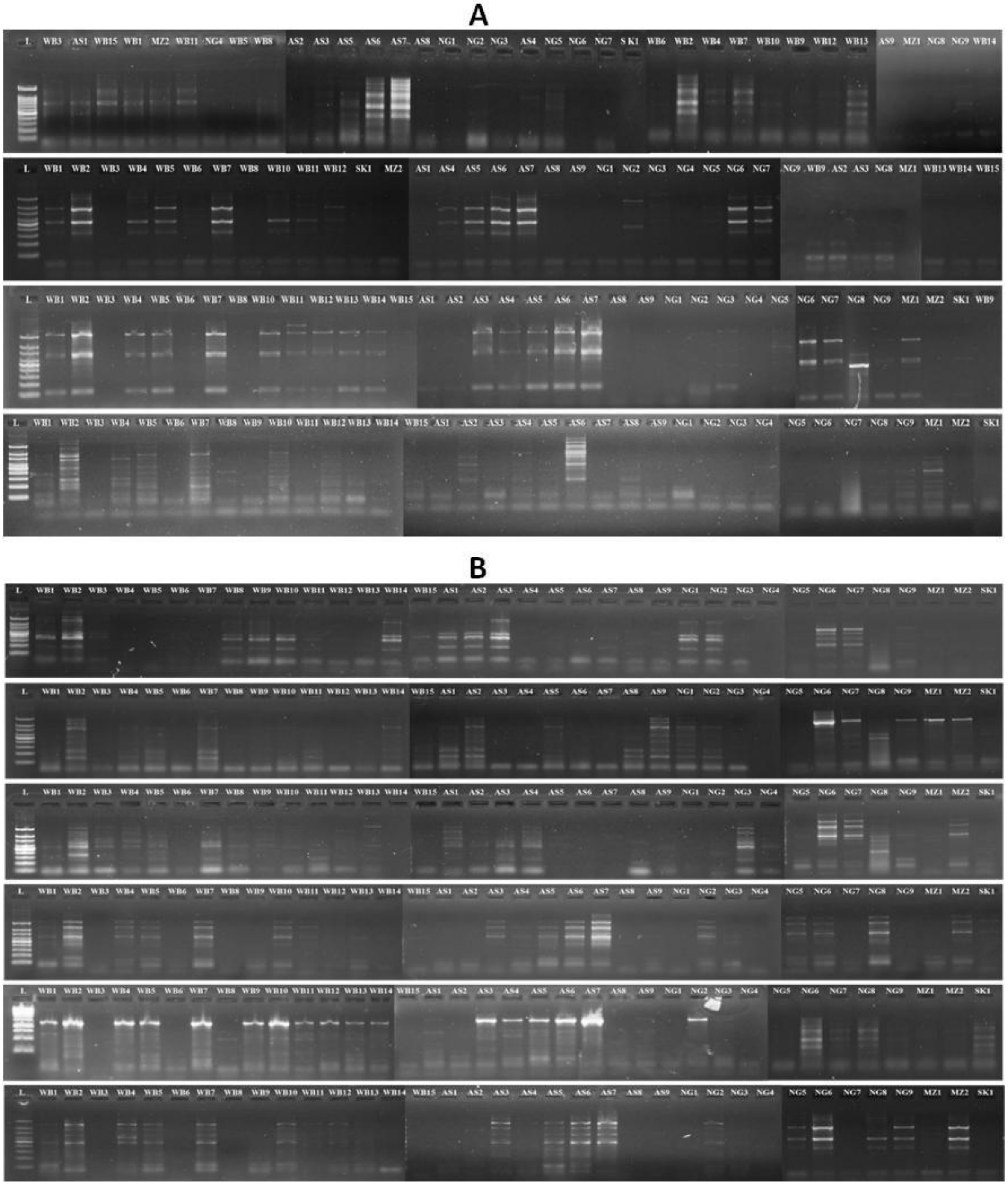

S3 Fig. DNA amplification of *S. sclerotiorum* with UP-PCR primers. A: AA2M2, AS4, 3-2 and L-21 primers (top to bottom); B: AS4 & 3-2, AA2M2 & AS4, AA2M2 & 3-2, 3-2 & L-21, AA2M2 & L-21 and AS4 & L-21 primers combination
