# Supplementary material for: Genetic diversity and virulence variability of Sclerotinia sclerotiorum in Eastern and Northeastern India
Source: PLoS One. 2024 Nov 25;19(11):e0312472. doi: 10.1371/journal.pone.0312472 (PMC11588274; doi:10.1371/journal.pone.0312472)
Supplement: S4 Fig — (PDF) [file pone.0312472.s011.pdf]

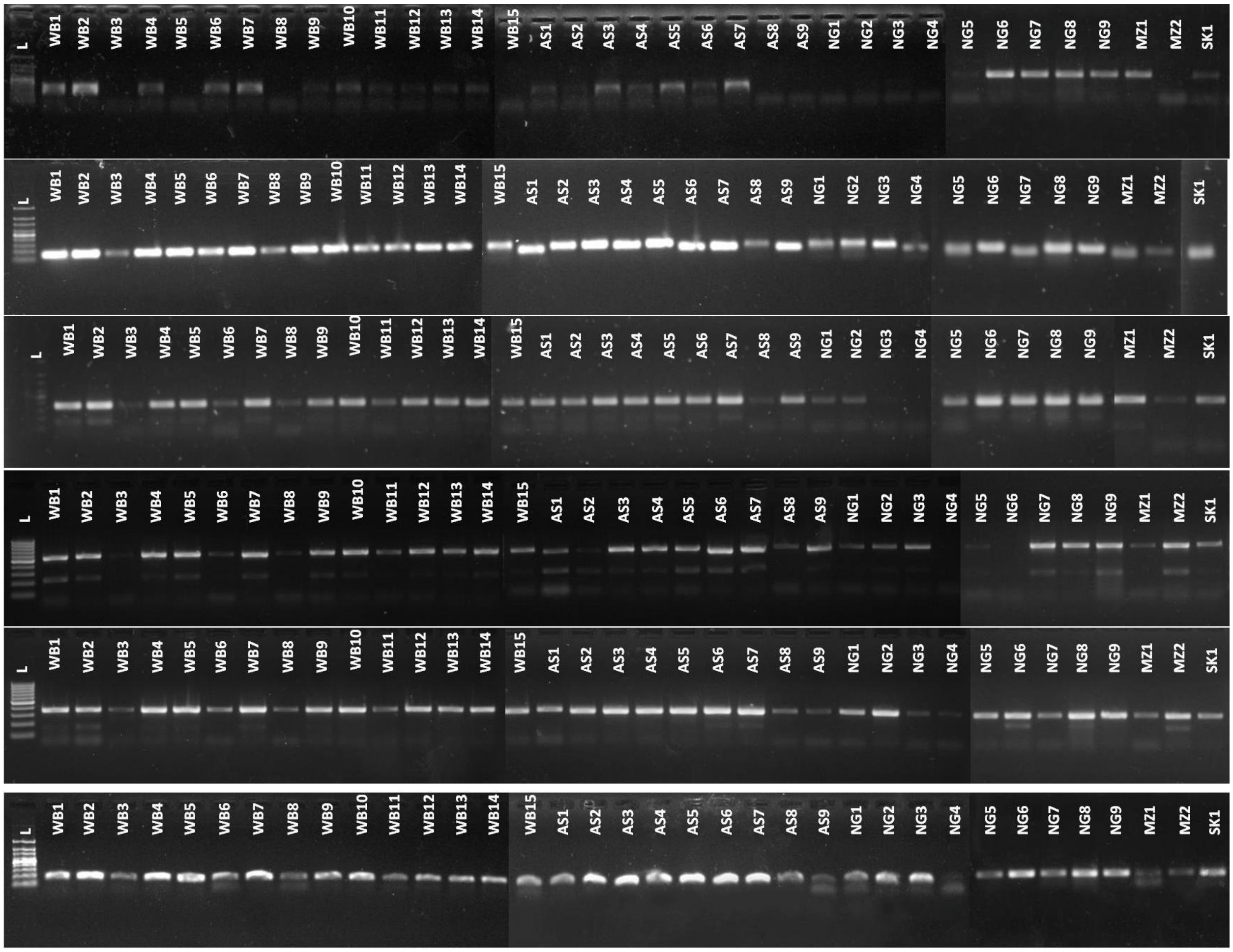

S4 Fig. DNA amplification of *S. sclerotiorum* isolates with microsatellite primers; $(GA)_{14}$, $(CA)_9$, $(TTA)_9$, $TACA_{10}$, $(CT)_{12}$, $(CATA)_{25}$ and $(AGAT)_{14}(AAGC)_4$ primers (top to bottom)
