# Supplementary material for: Genetic diversity and virulence variability of Sclerotinia sclerotiorum in Eastern and Northeastern India
Source: PLoS One. 2024 Nov 25;19(11):e0312472. doi: 10.1371/journal.pone.0312472 (PMC11588274; doi:10.1371/journal.pone.0312472)

**Note: The ladder (L) used for the study is of 100bp**

**S1 Fig. Amplification of *S. sclerotiorum* genomic DNA with ITS primers**

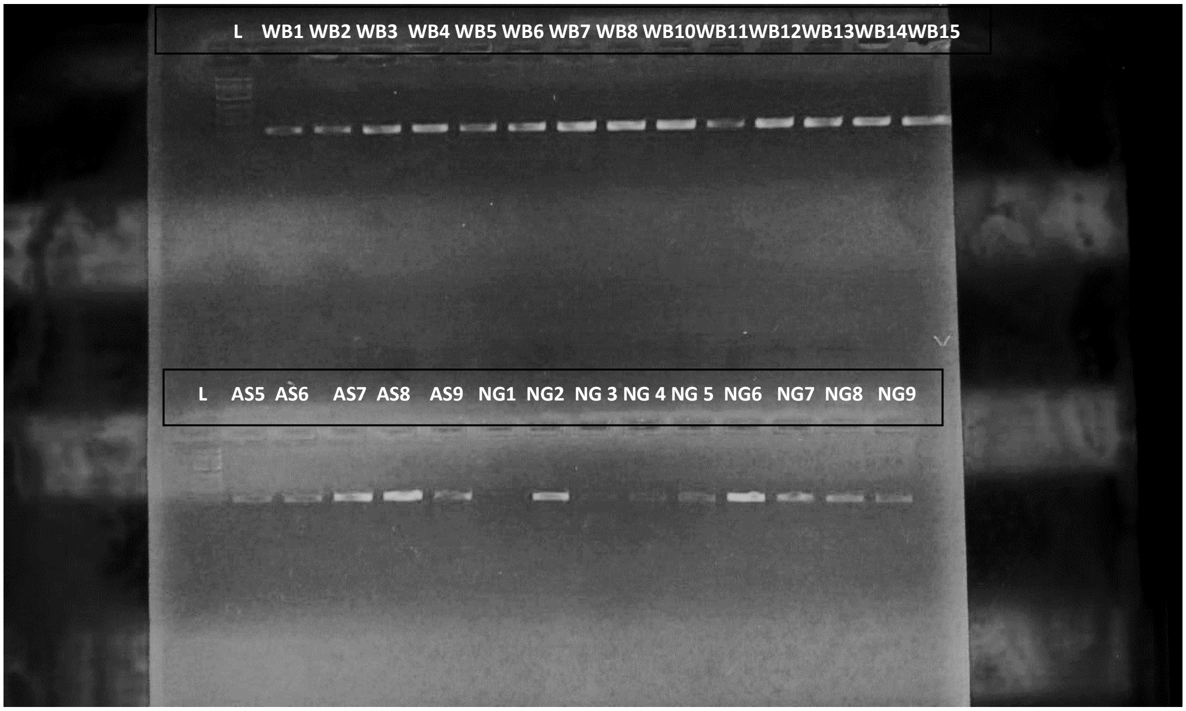


**S3Fig. DNA amplification of *S. sclerotiorum* with UP-PCR primers. Gel A: AA2M2(Row 1)**

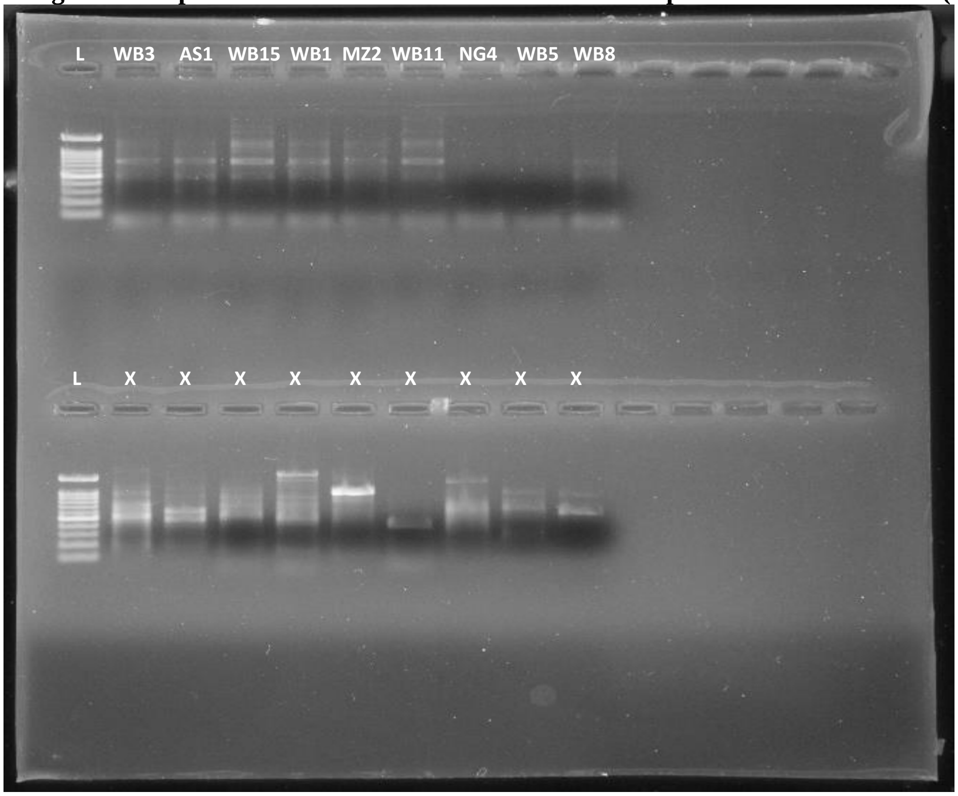

Gel A: AA2M2 (Row 1)

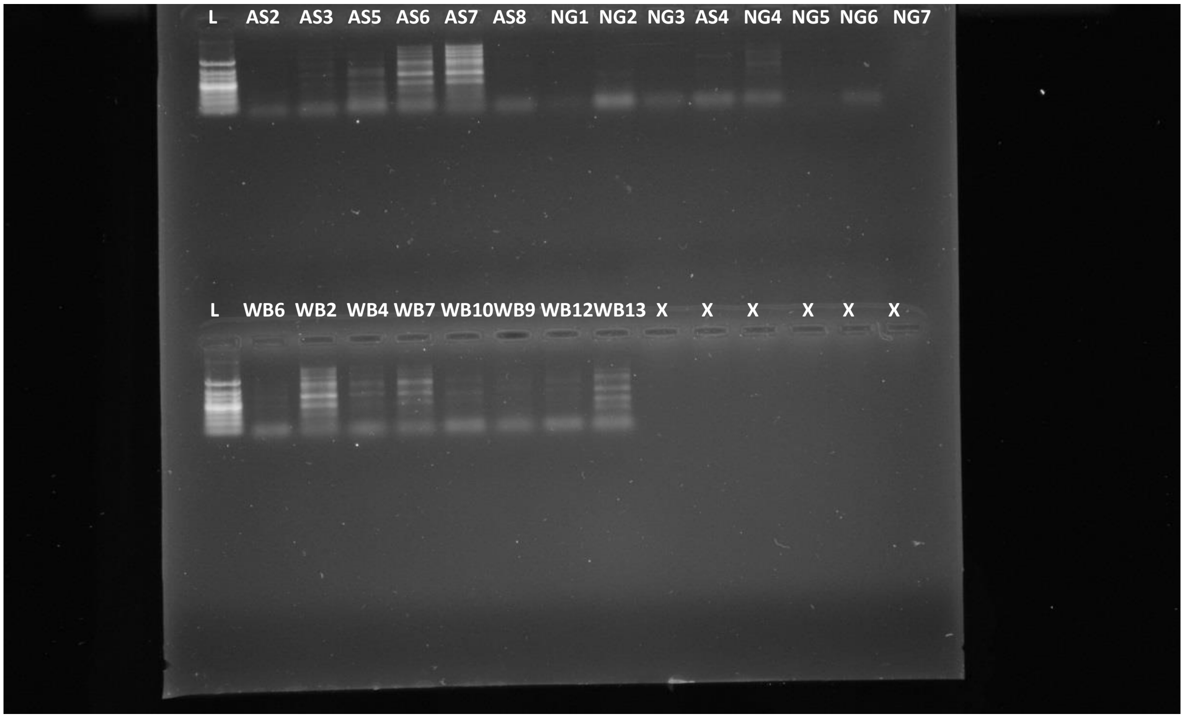


S3Fig. Gel A – AS4 (Row 2)

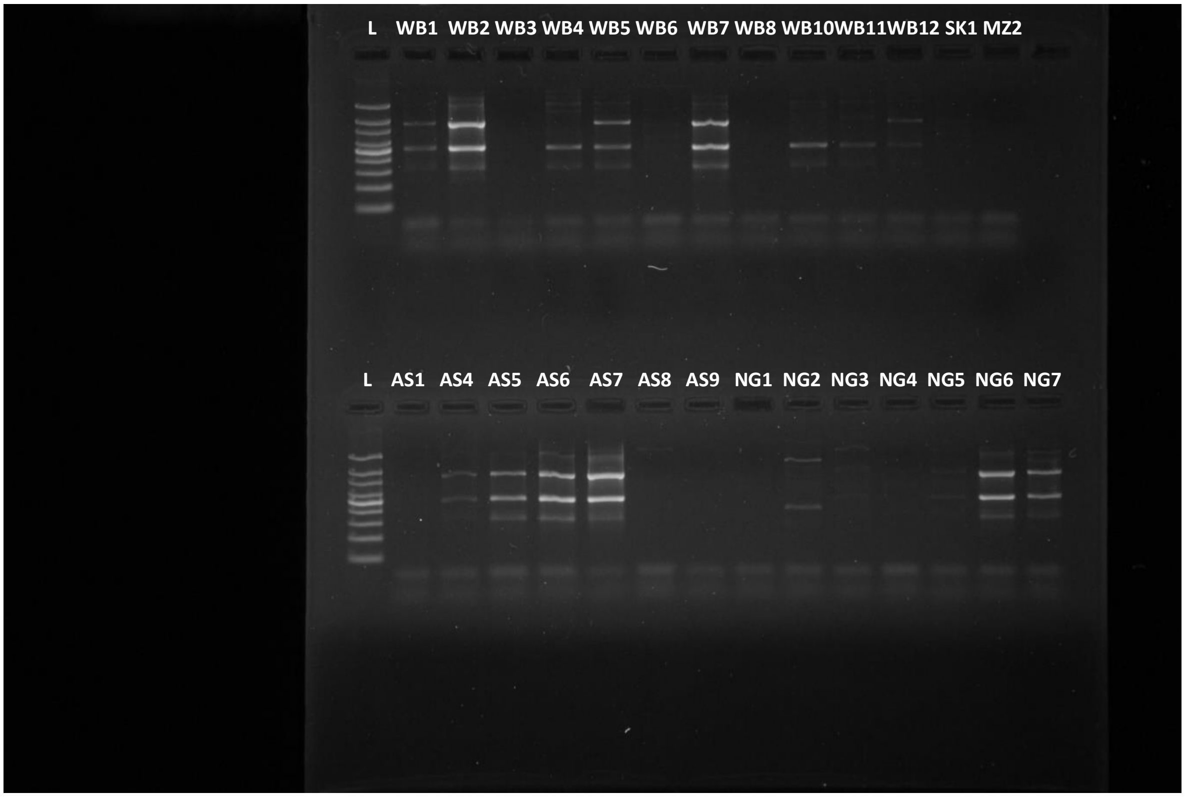

S3Fig. Gel A – AA2M2 (Row 1) & AS4 (Row 2)

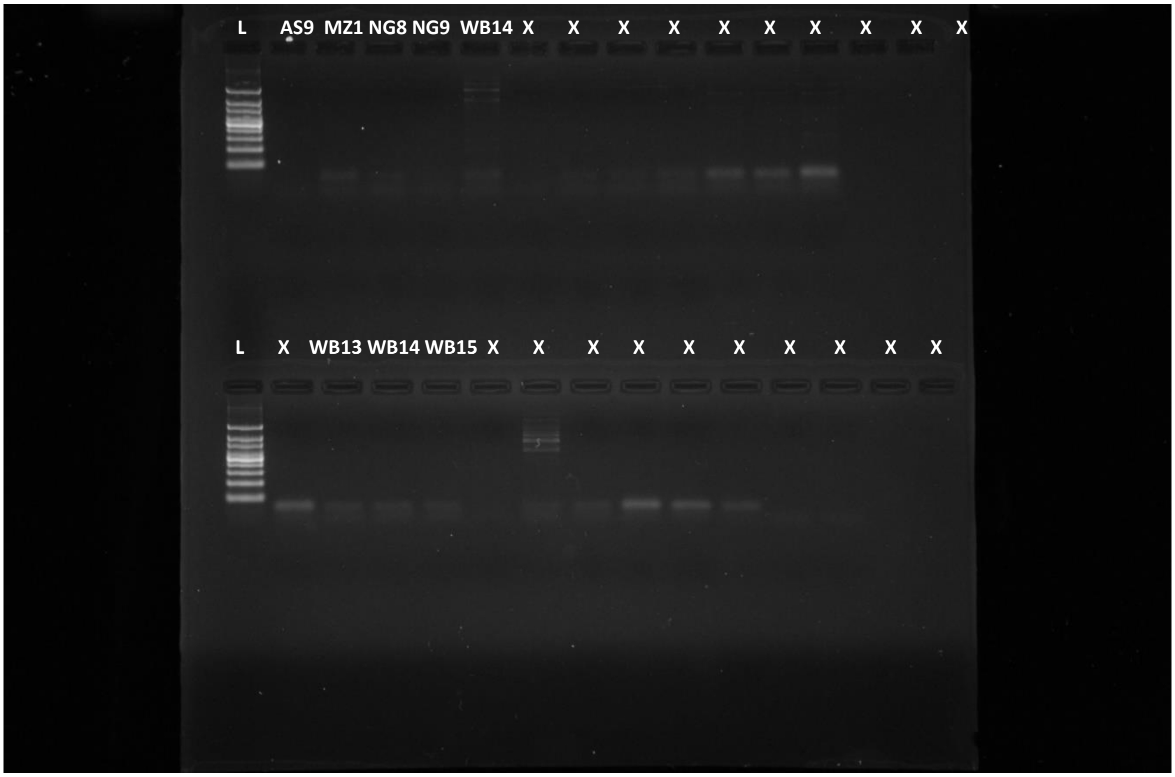


S3Fig. Gel A – 3-2(Row 3)

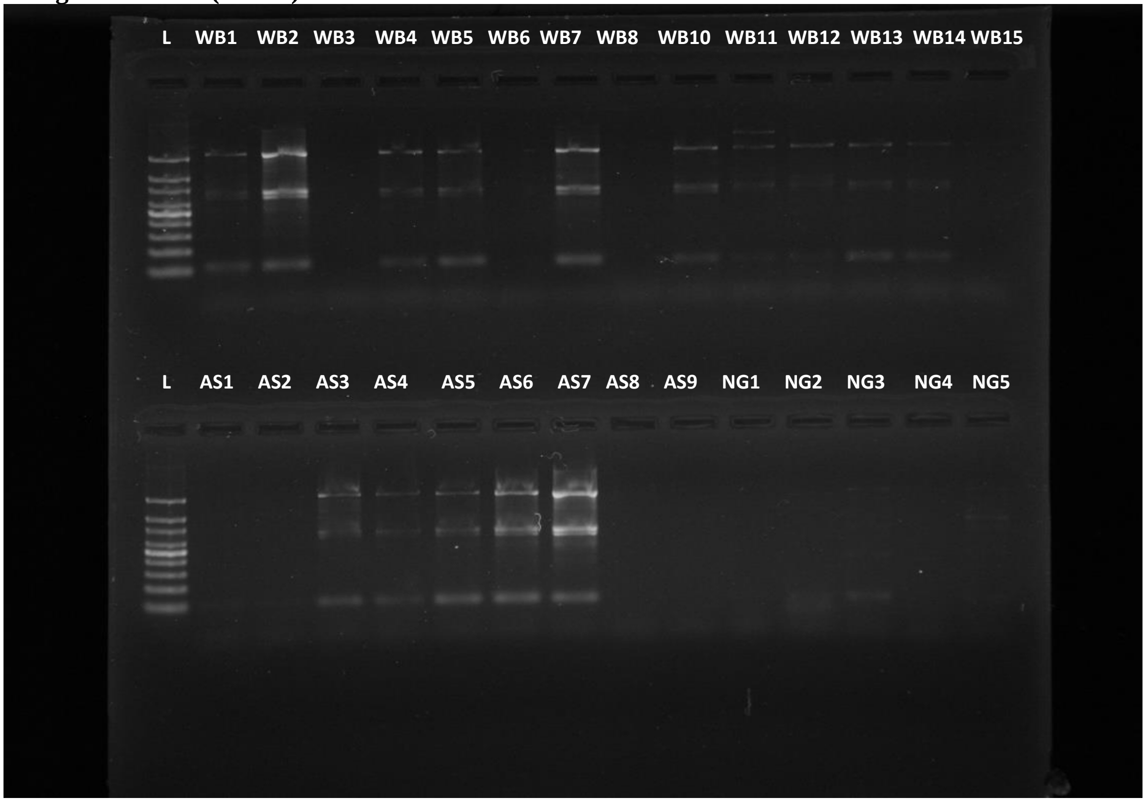

**S3Fig. Gel A – L-21(Row 4)**

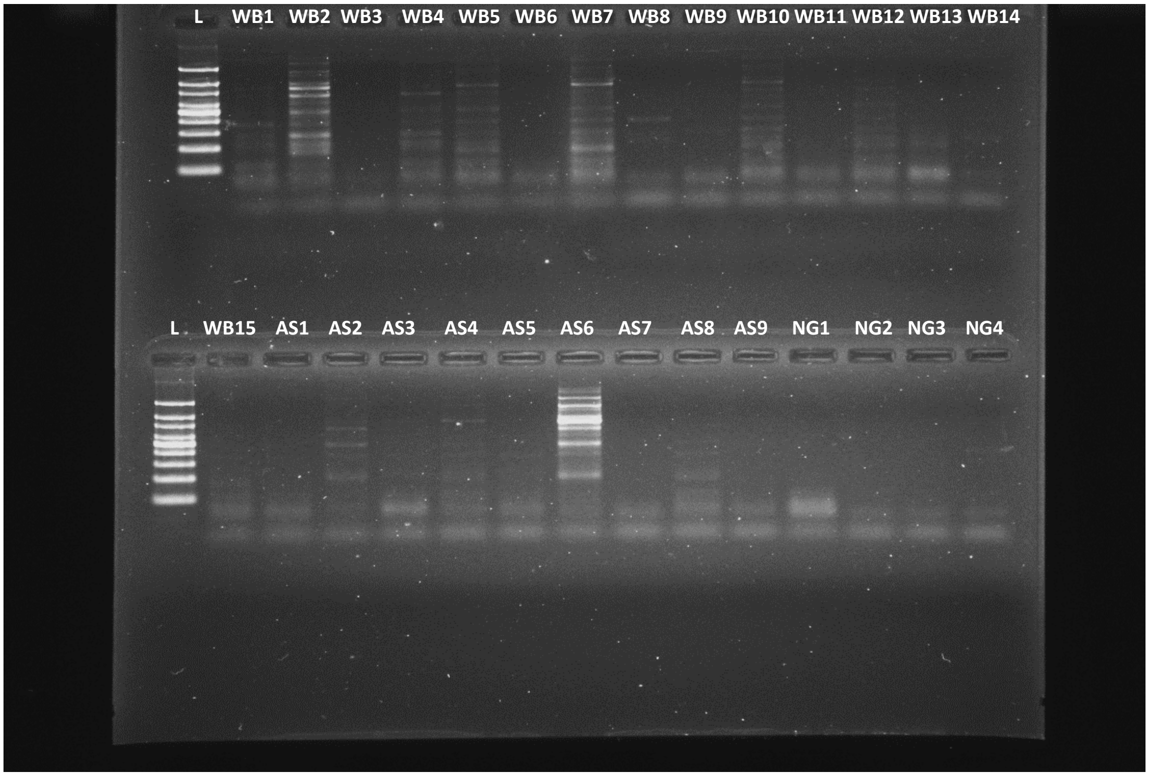


**S3Fig. Gel A – 3-2(Row 3) & L-21(Row 4)**

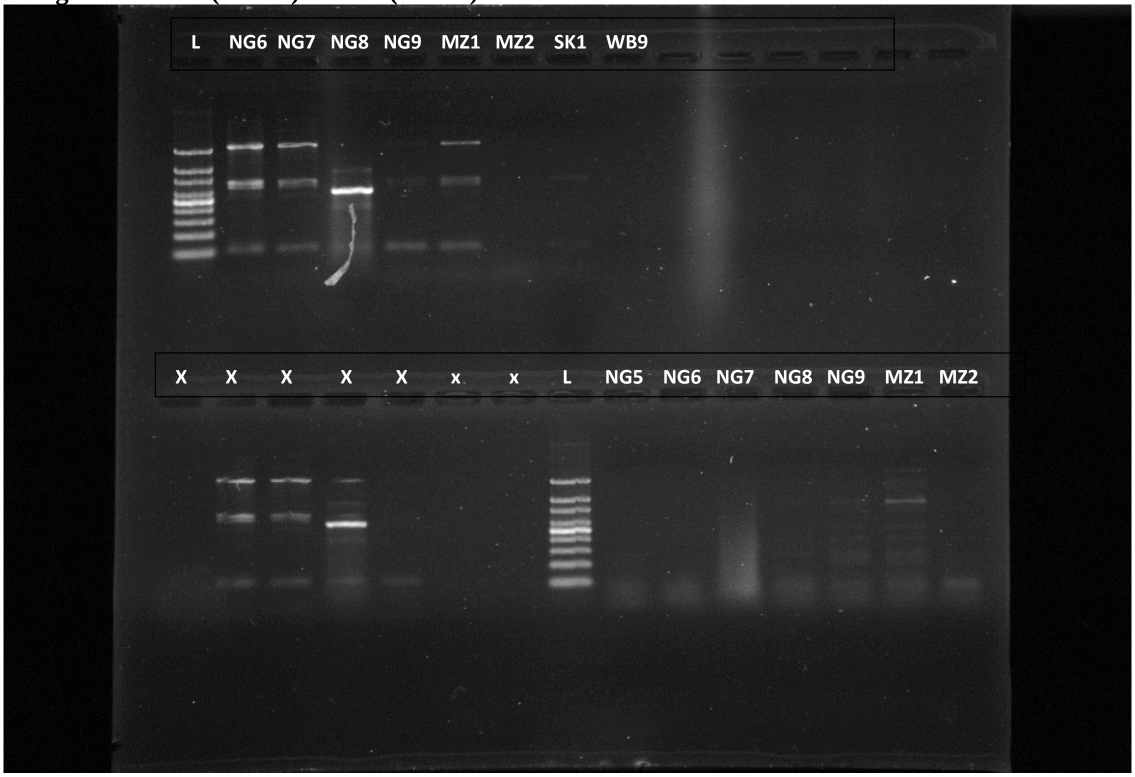

**S3 Fig. Gel B – AS4 & 3-2(Row 1)**

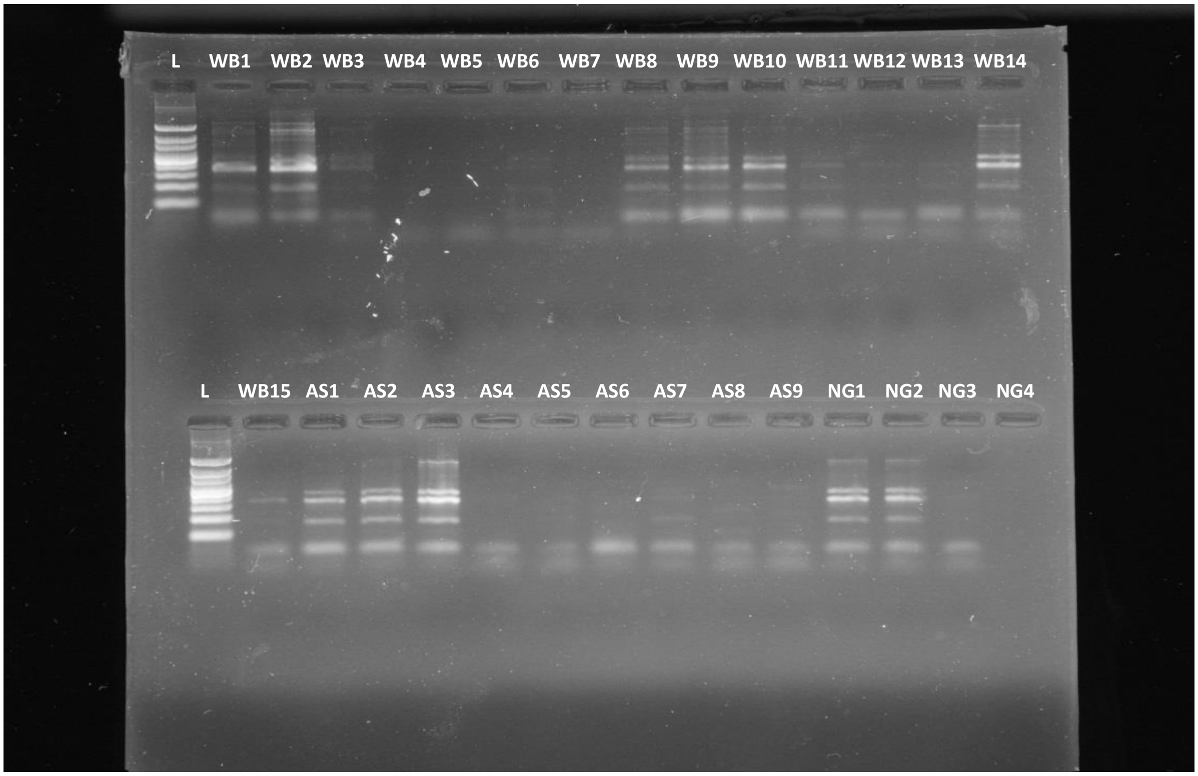


**S3Fig. Gel B – AS4 & 3-2(Row1)**

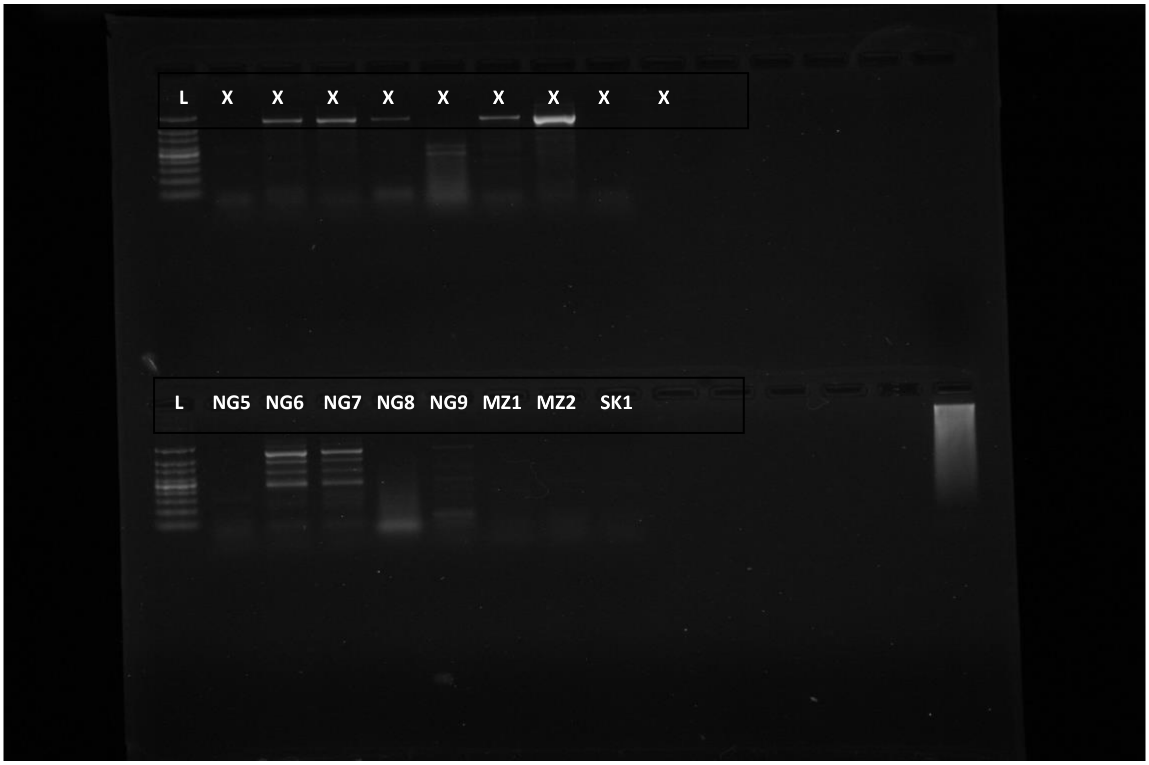

S3Fig. Gel B – AA2M2 & AS4 (Row 2)

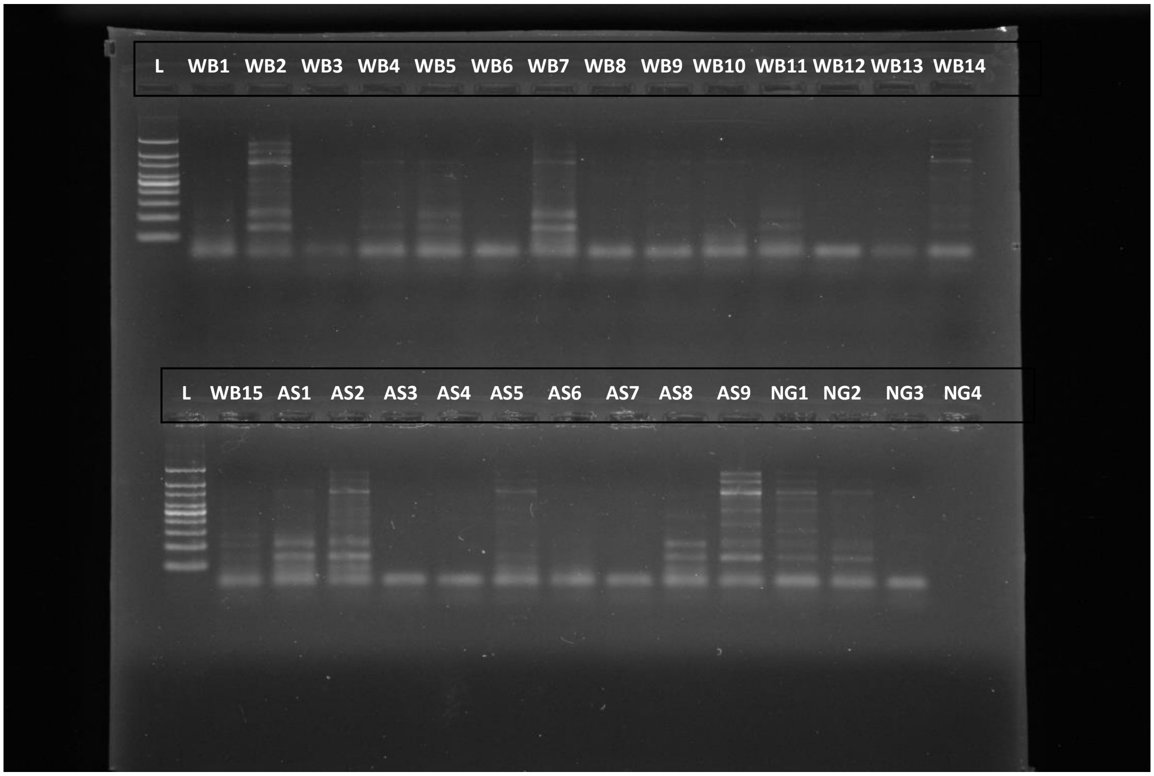


S3Fig. Gel B – AA2M2 & 3-2(Row 3)

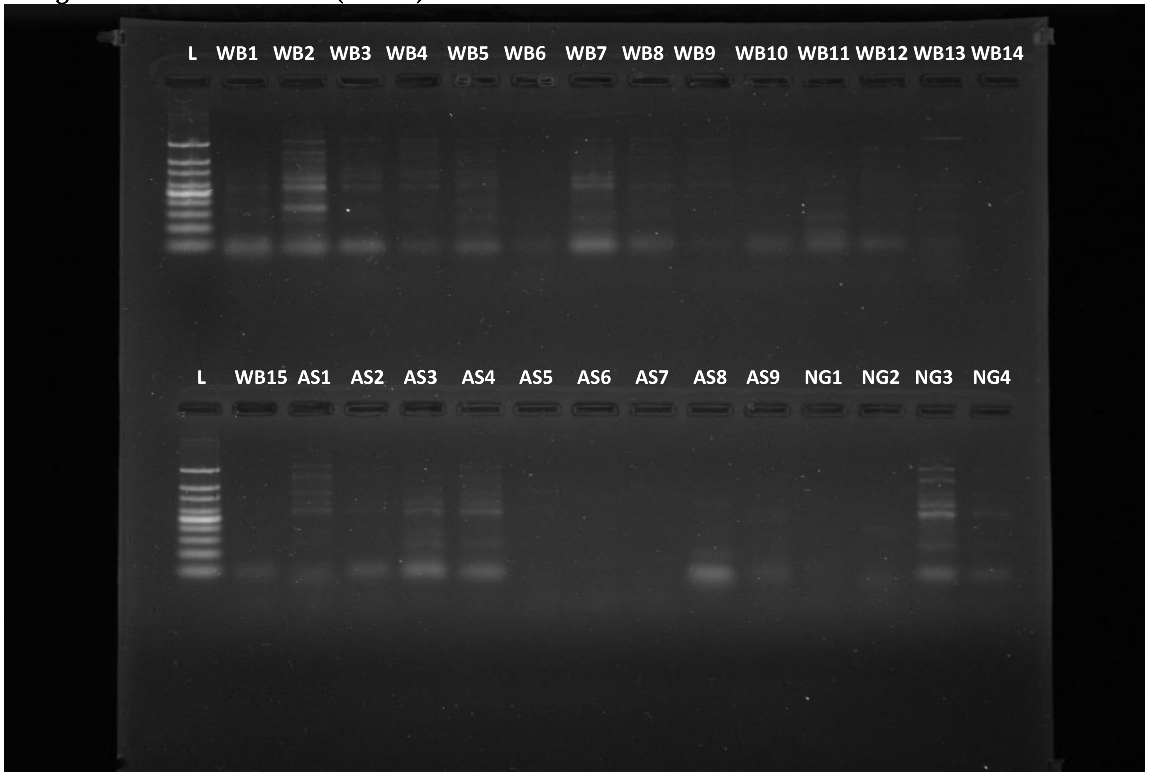

S3Fig. Gel B – AA2M2 & AS4(Row2) – AA2M2 & 3-2(Row 3)

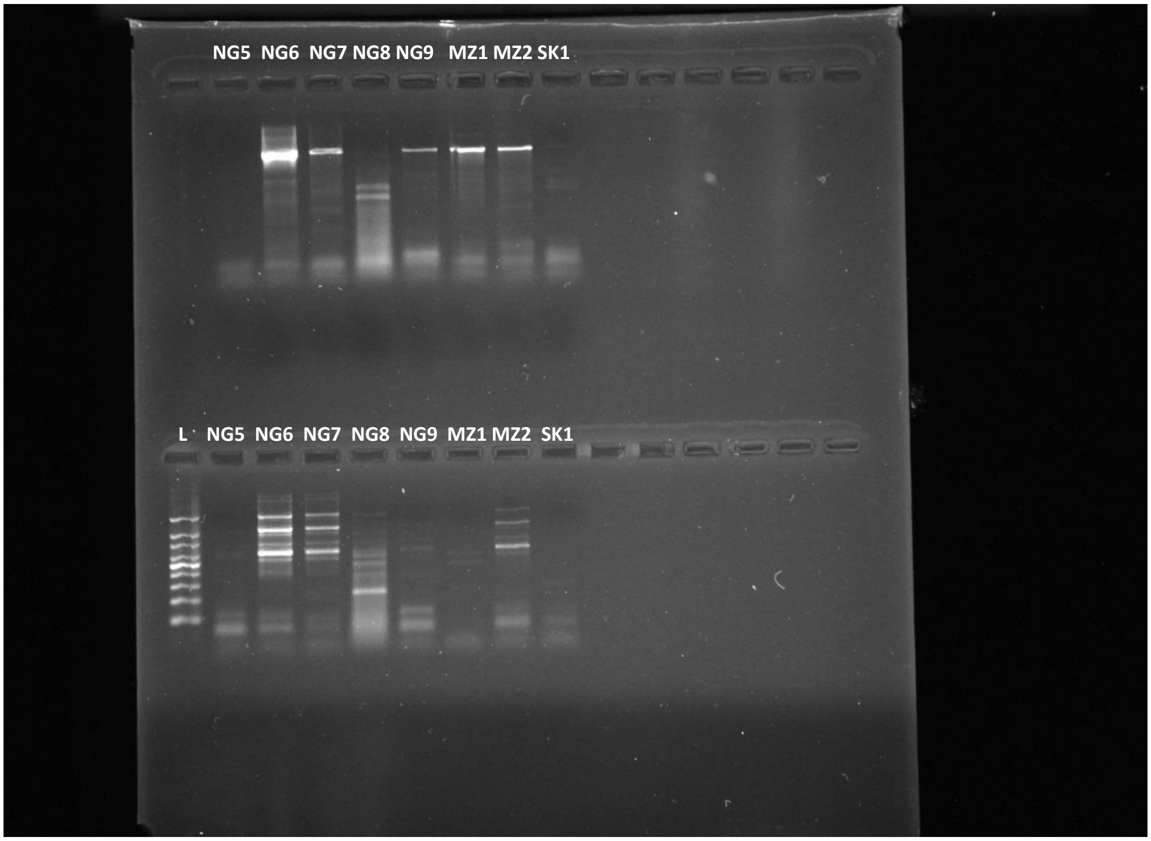


S3Fig. Gel B – 3-2 & L-21 (Row 4)

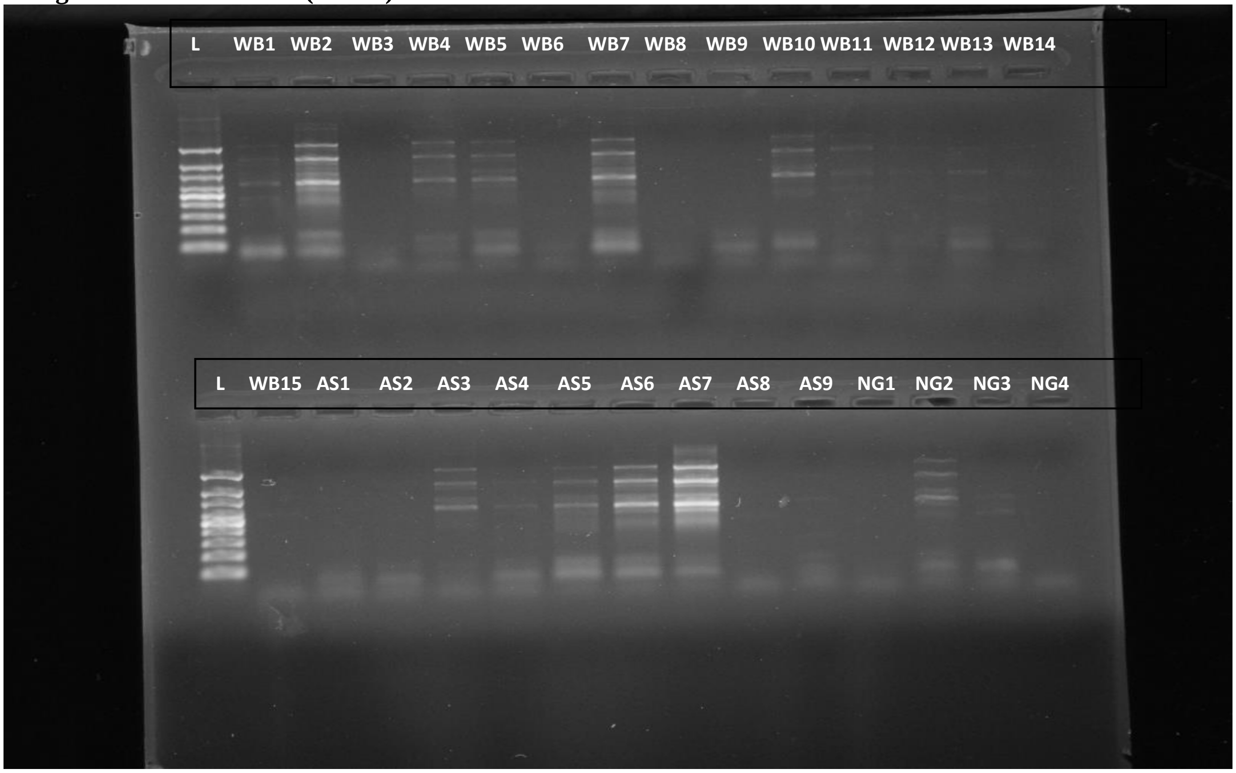

**S3 Fig. Gel B – AA2M2 & L21(Row 5)**

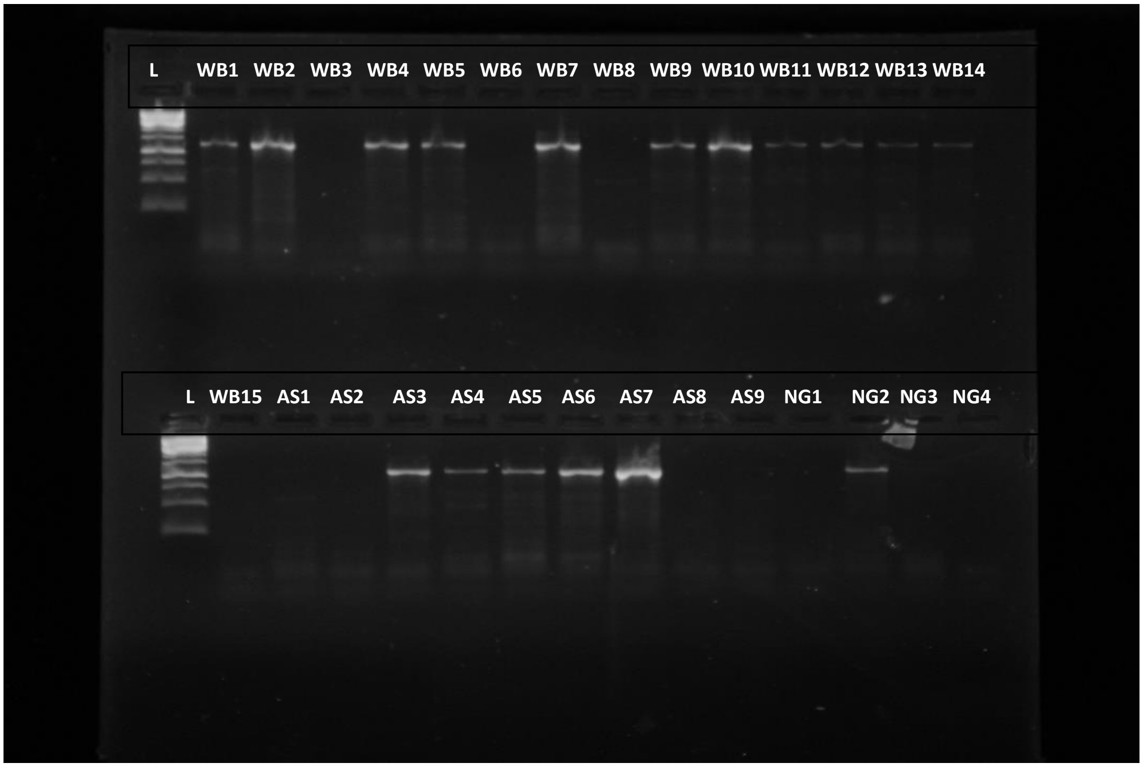


**S3Fig. Gel B – 3-2 & L-21 (Row 4) &AA2M2 & L21 (Row 5)**

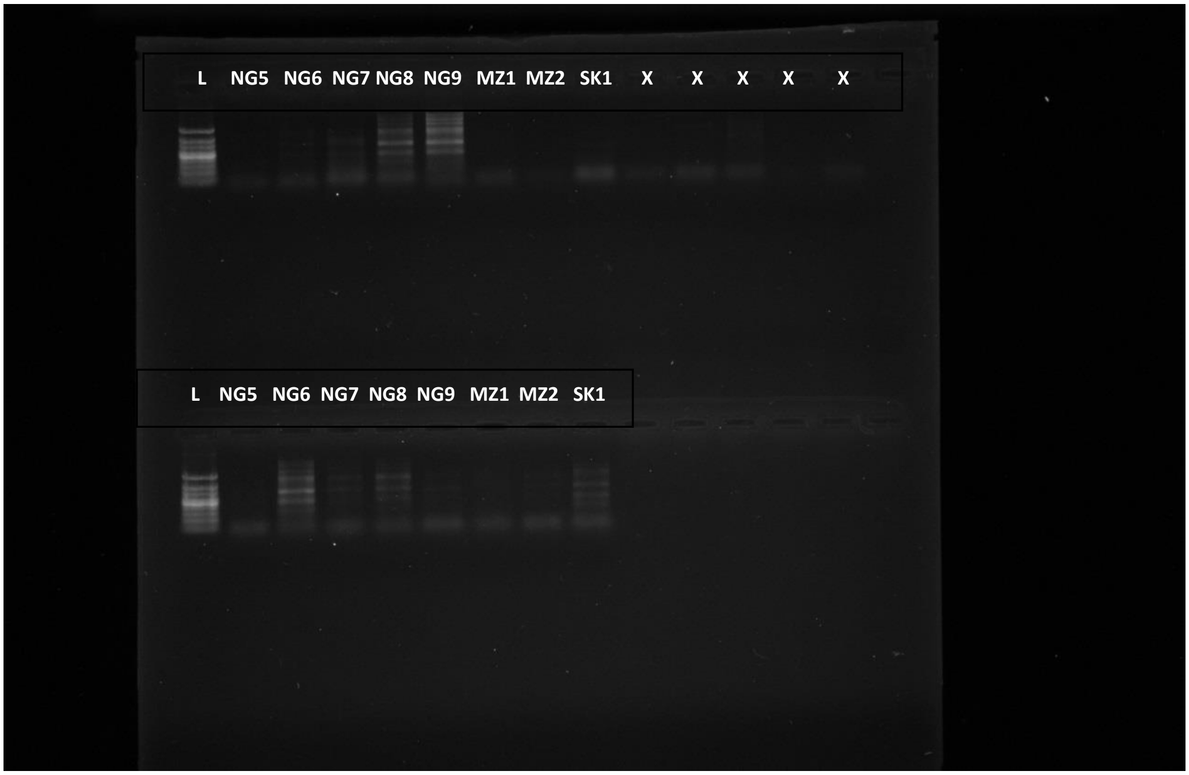

**S3 Fig. Gel B – AS4 & L-21 (Row 6)**

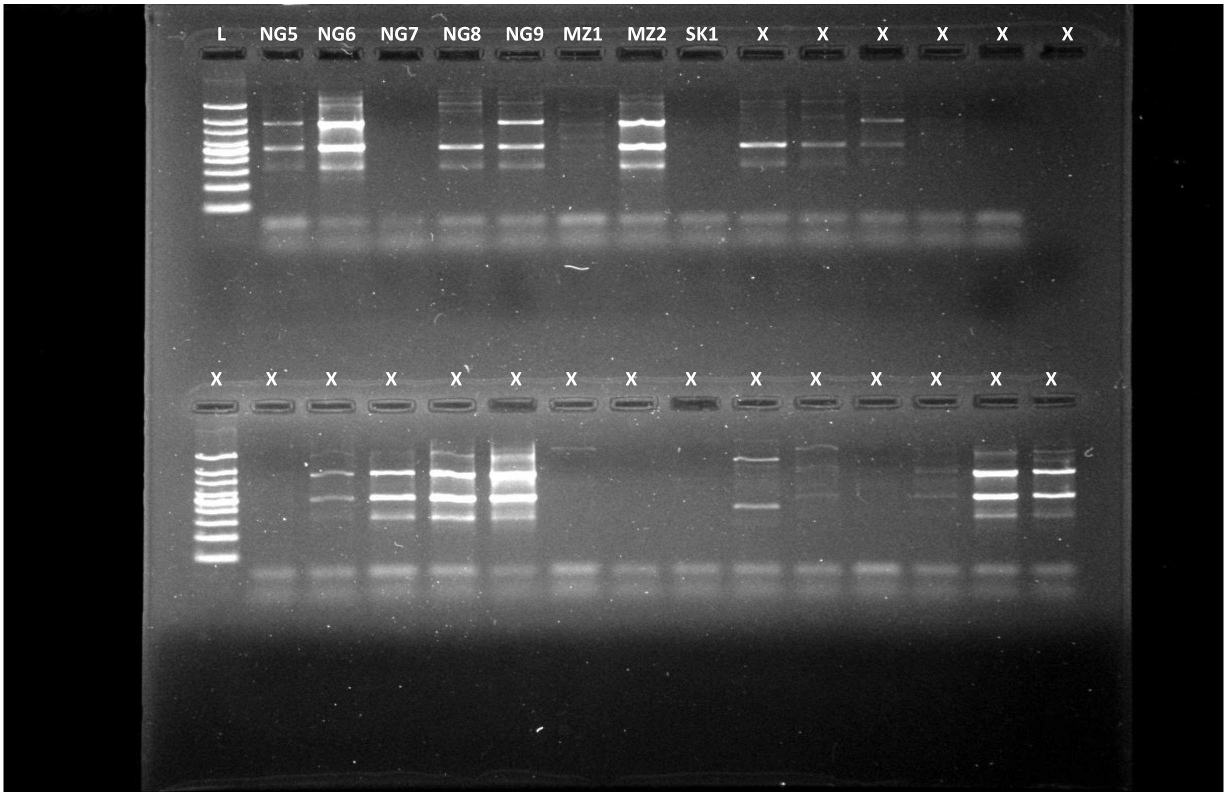


**S3 Fig. Gel B – AS4 & L-21 (Row 6)**

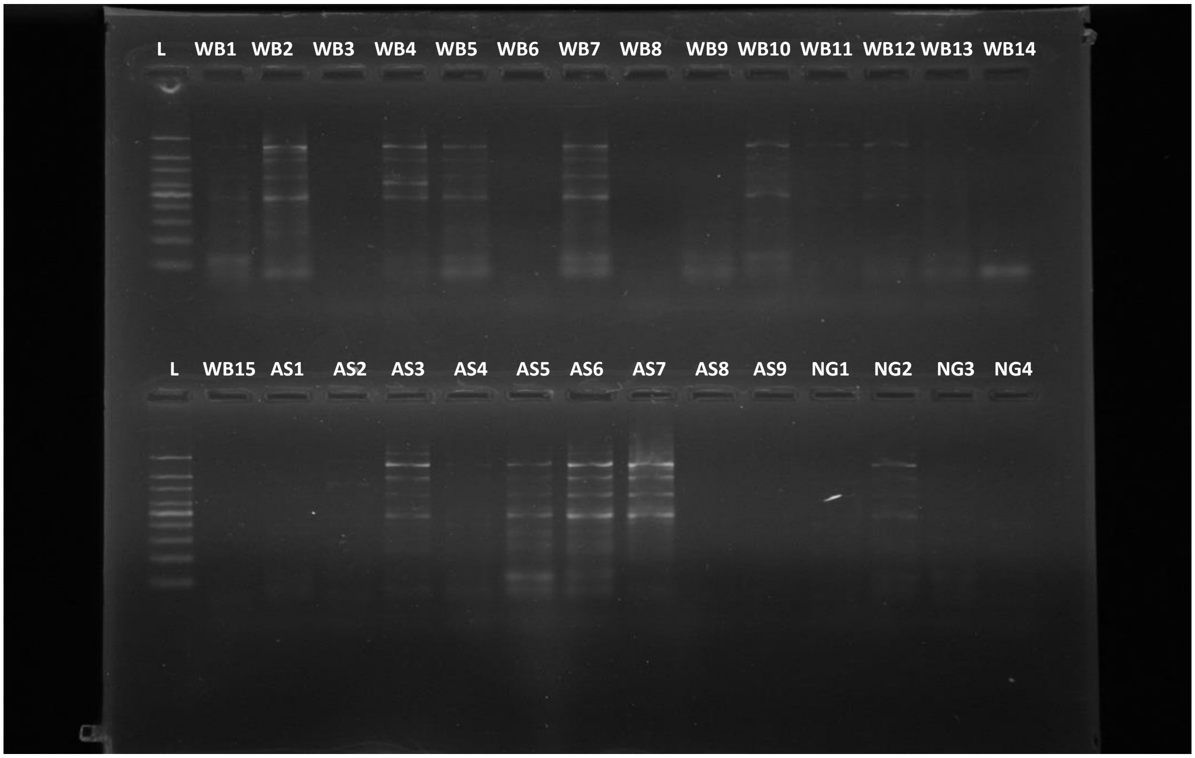

**S4 Fig. DNA amplification of *S. sclerotiorum* isolates with microsatellite; $(GA)_{14}$ Row 1**

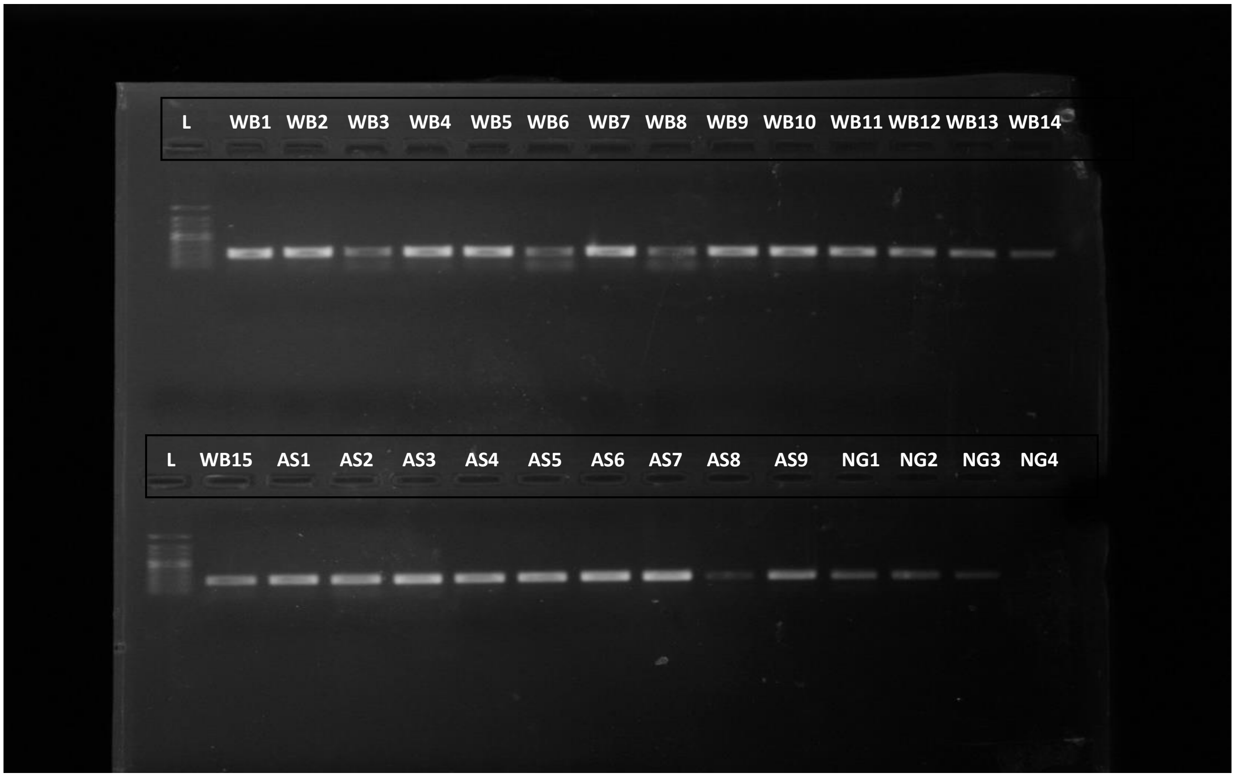


**S4 Fig. – (CA)9 Row 2**

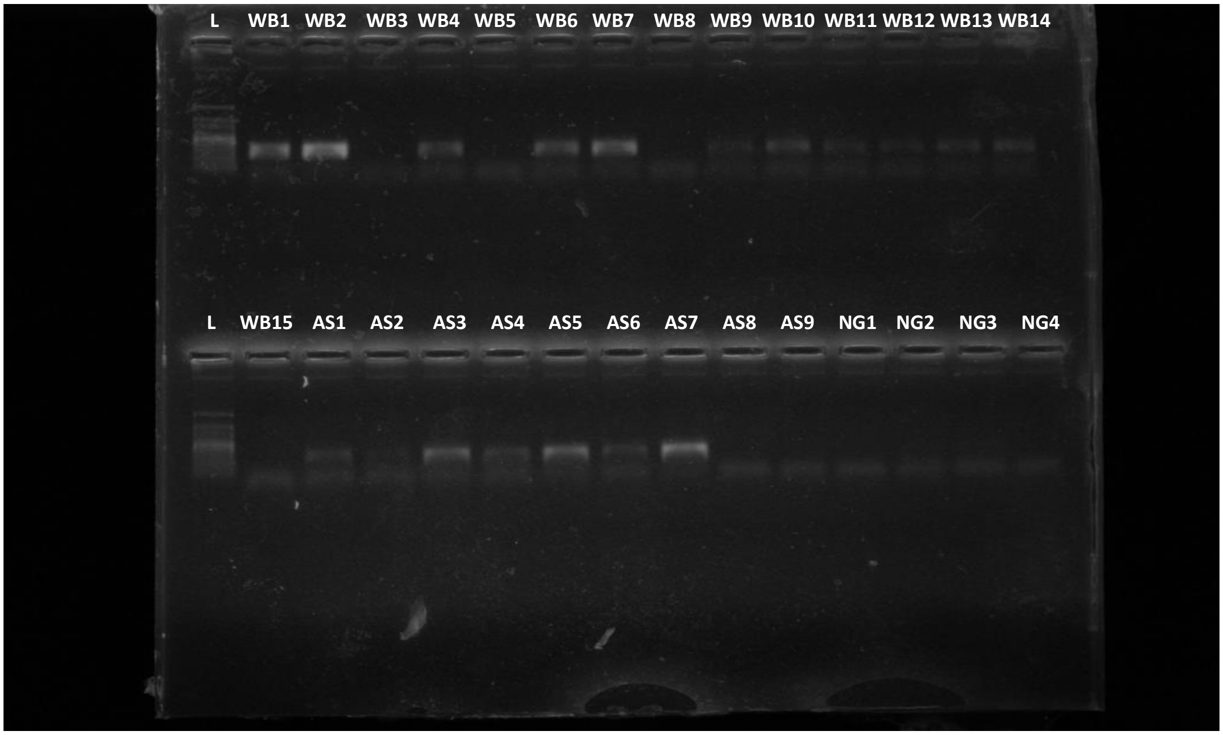

**S4 Fig. – $(GA)_{14}$ Row 1 & $(CA)_9$ Row 2**

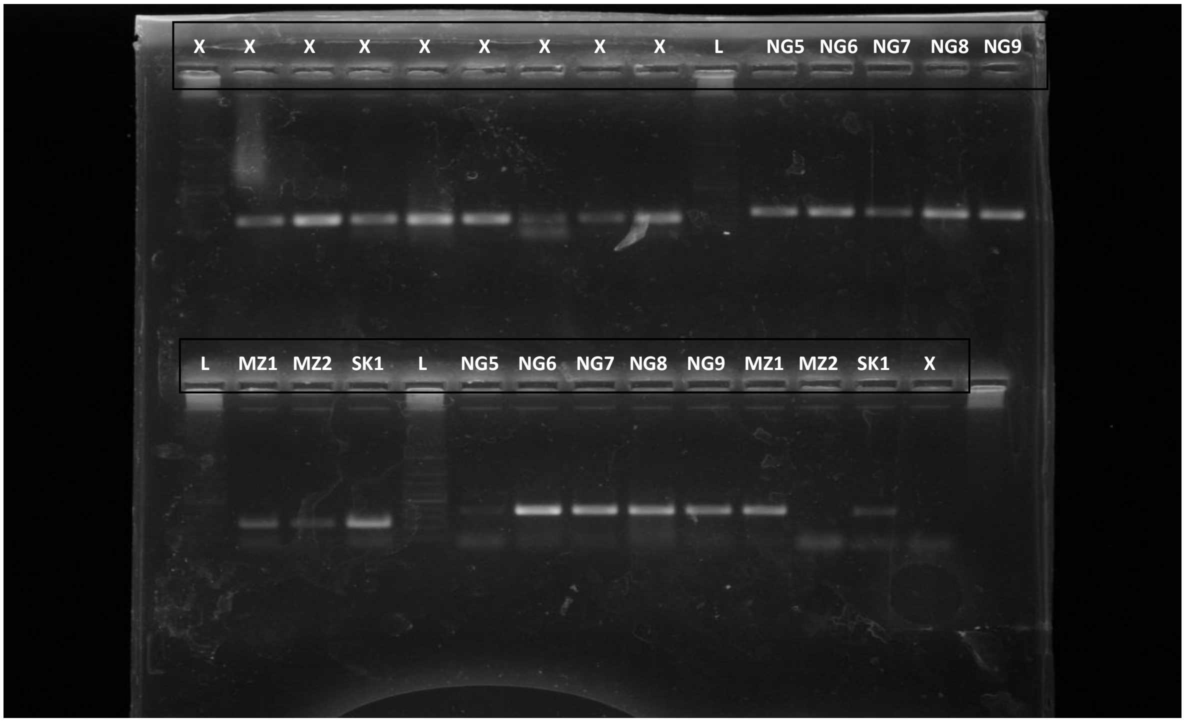


**S4 Fig. – $(TTA)_9$ Row 3**

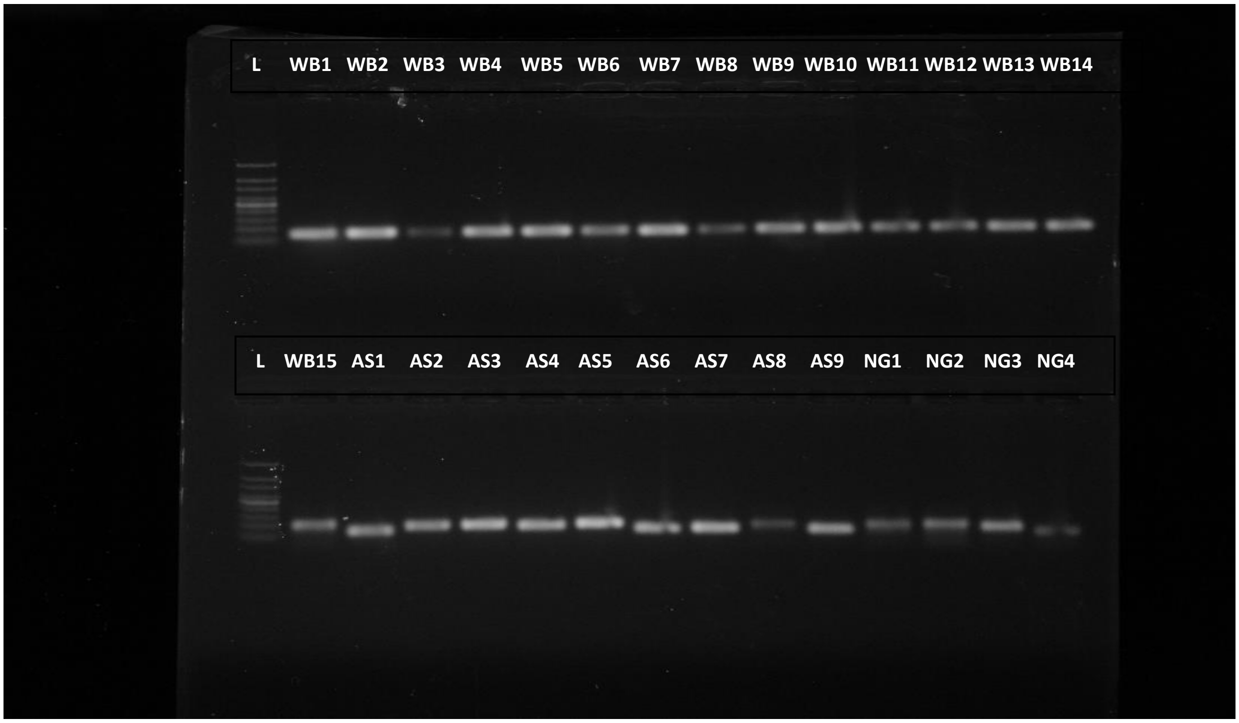

**S4 Fig. – $TACA_{10}$ Row 4**

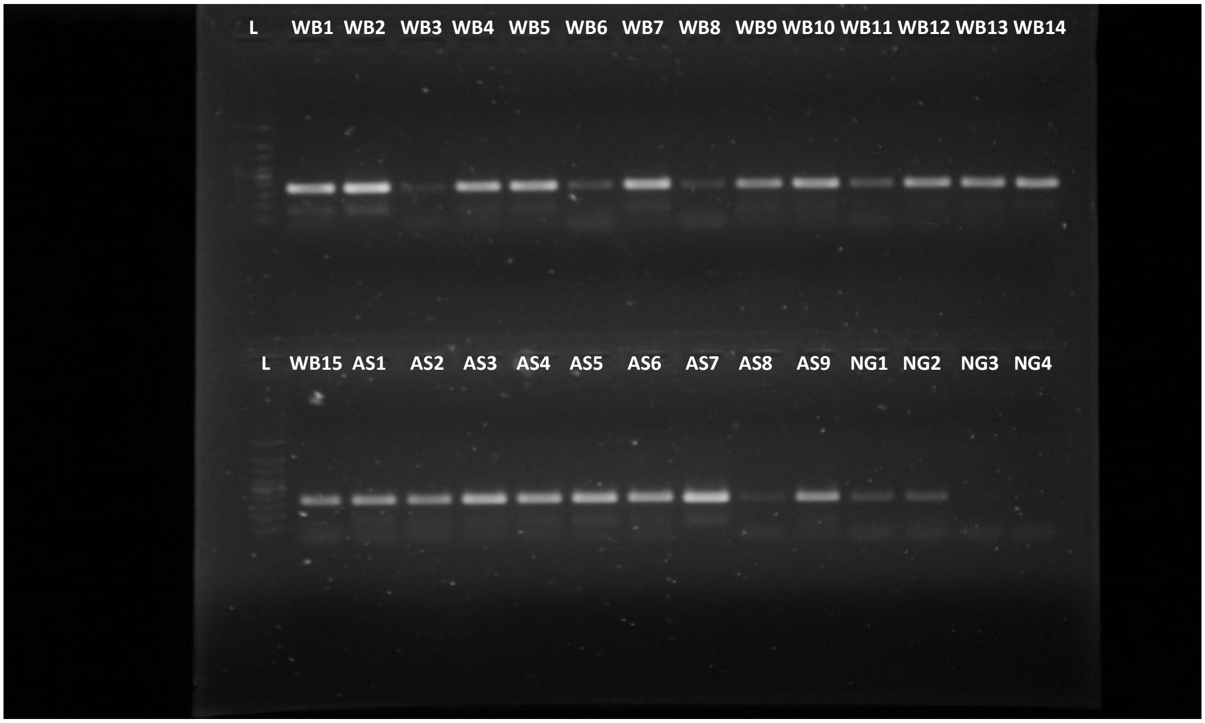


**S4 Fig. – $(TTA)_9$ Row 3 & $TACA_{10}$ Row 4**

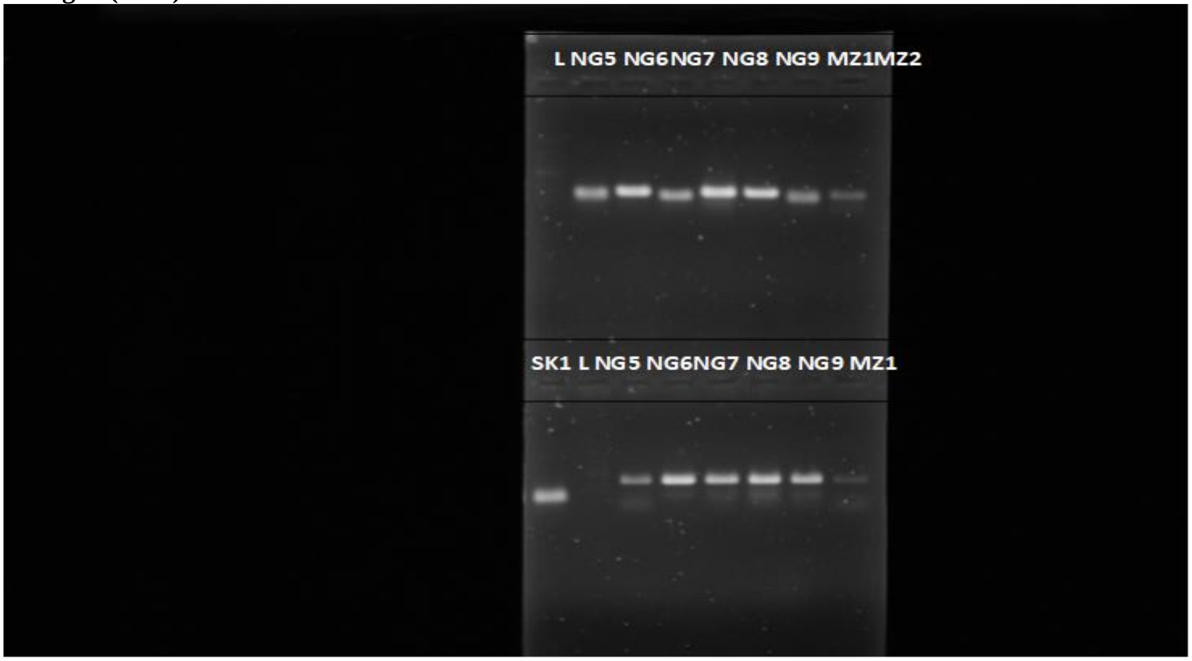

**S4 Fig. – (CT)$_{12}$ Row 5**

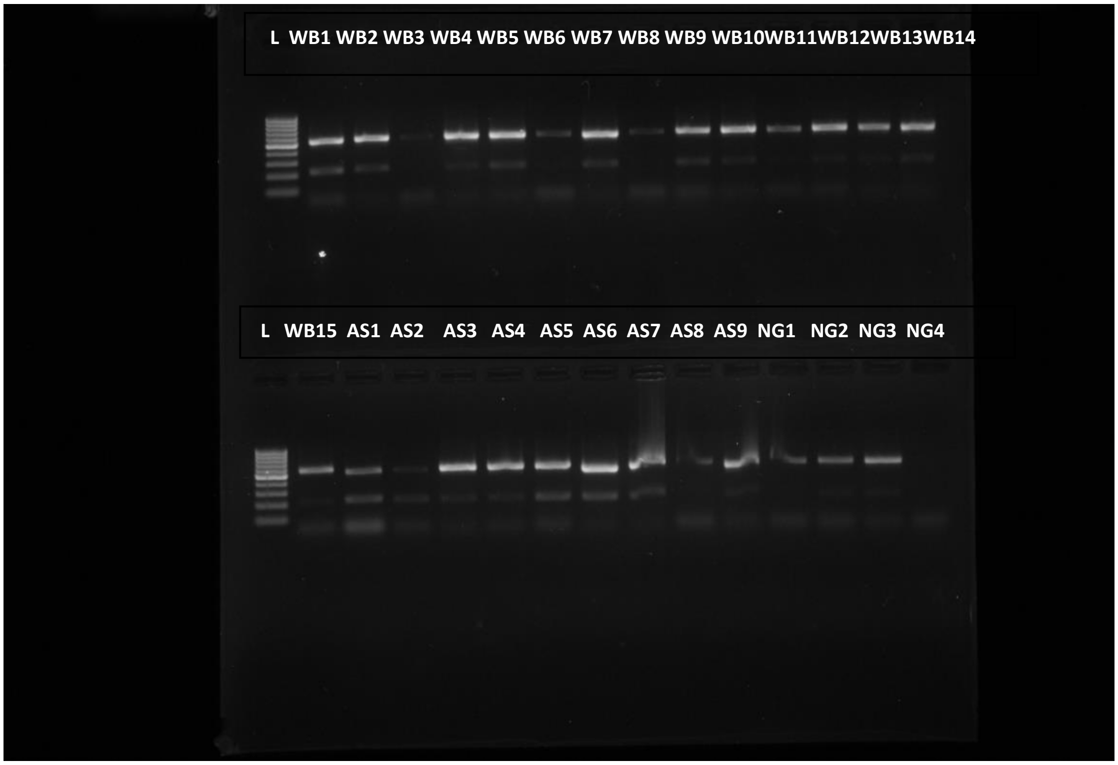


**S4 Fig. – (CATA)$_{25}$ Row 6**

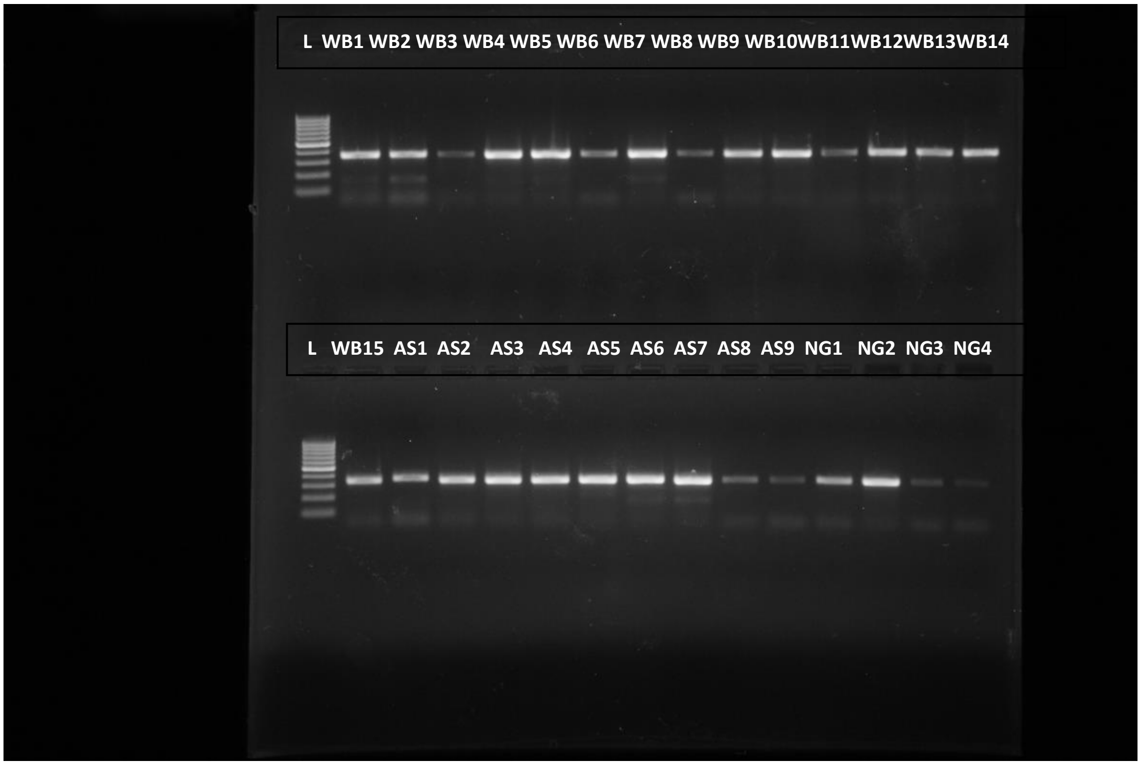

S4 Fig. –$(CT)_{12}$ Row 5 & $TACA_{10}$ Row 4 & $(CATA)_{25}$ Row 6

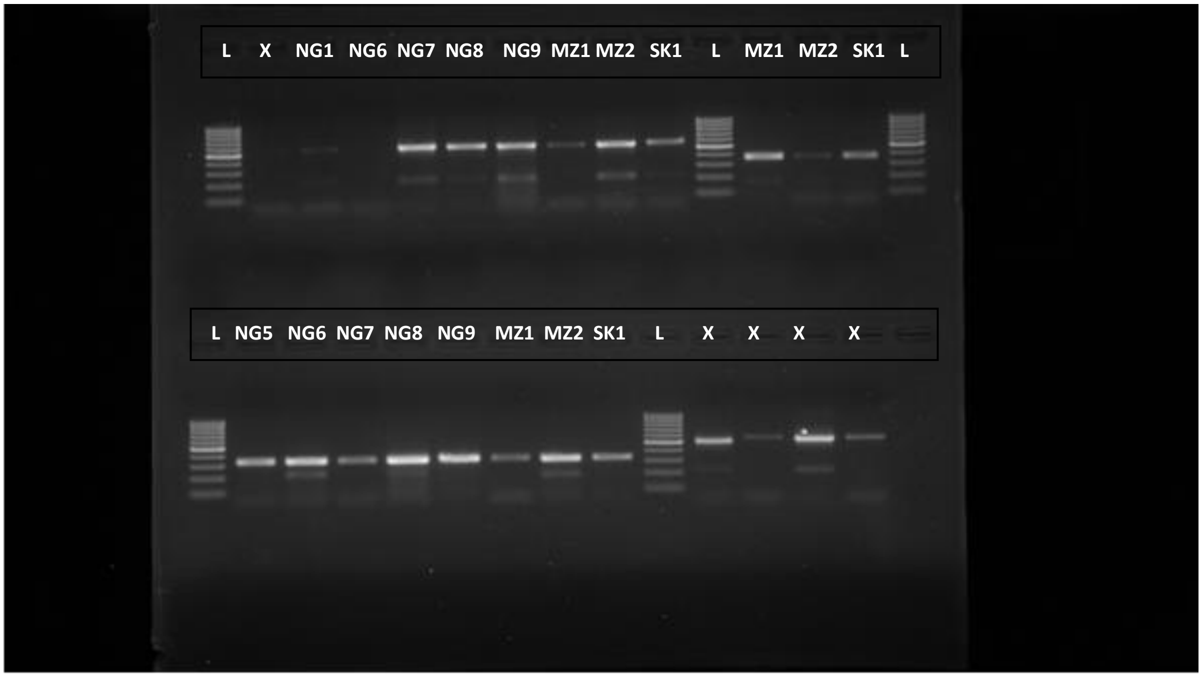


S4 Fig. - $(AGAT)_{14}(AAGC)_4$ Row 7

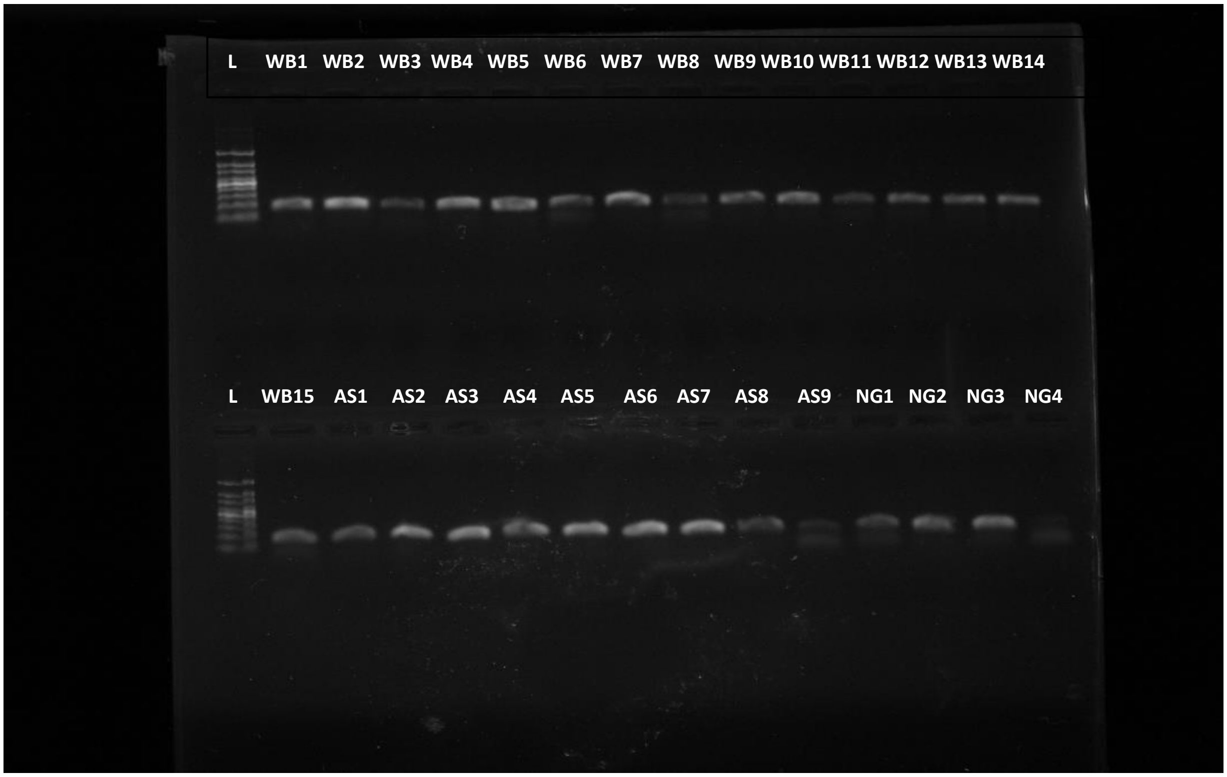

**S4 Fig. - $(AGAT)_{14}(AAGC)_4$ Row 7**

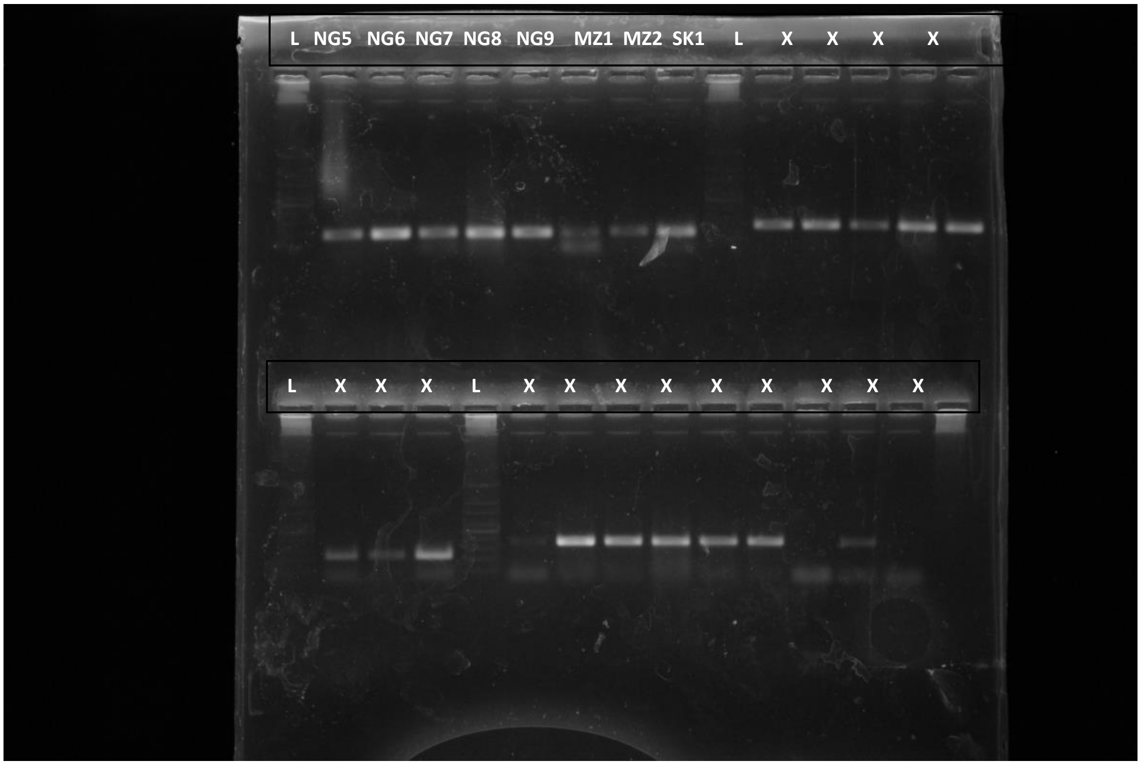

Supplement: S1 Raw image — (PDF) [file pone.0312472.s012.pdf]
